# Impact of neighbourhood and environmental factors on the risk of incident cardiovascular disease: a systematic review and meta-analysis

**DOI:** 10.1093/eurjpc/zwaf165

**Published:** 2025-03-19

**Authors:** Jack R G Brown, Paris J Baptiste, Hajar Hajmohammadi, Ramesh Nadarajah, Chris P Gale, Jianhua Wu

**Affiliations:** Centre for Primary Care, Wolfson Institute of Population Health, Queen Mary University of London, London, E1 2AT, UK; Centre for Primary Care, Wolfson Institute of Population Health, Queen Mary University of London, London, E1 2AT, UK; Centre for Primary Care, Wolfson Institute of Population Health, Queen Mary University of London, London, E1 2AT, UK; Leeds Institute of Data Analytics, University of Leeds, Leeds, LS2 9NL, UK; Leeds Institute for Cardiovascular and Metabolic Medicine, University of Leeds, Leeds, LS2 9NL, UK; Department of Cardiology, Leeds Teaching Hospitals NHS Trust, Leeds, LS1 3EX, UK; Leeds Institute of Data Analytics, University of Leeds, Leeds, LS2 9NL, UK; Leeds Institute for Cardiovascular and Metabolic Medicine, University of Leeds, Leeds, LS2 9NL, UK; Department of Cardiology, Leeds Teaching Hospitals NHS Trust, Leeds, LS1 3EX, UK; Centre for Primary Care, Wolfson Institute of Population Health, Queen Mary University of London, London, E1 2AT, UK

**Keywords:** Cardiovascular disease, Neighbourhood factors, Environmental exposures, Air pollution, Socioeconomic deprivation, Systematic review, Meta-analysis

## Abstract

**Aims:**

We aimed to study the association of five key neighbourhood exposures in large cohort studies and risk of incident cardiovascular disease (CVD).

**Methods and results:**

We conducted a systematic search of MEDLINE, The Cochrane Library, Web of Science, and Embase from database inception to 20th October 2024. Included studies reported both incident (first-time) CVD diagnosis and neighbourhood exposures across five domains: retail environment, health services, physical environment, pollution, and neighbourhood deprivation. A random-effects meta-analysis was performed to estimate pooled risk of CVD across domains. Of 39 studies included in the systematic review, 28 qualified for meta-analysis representing over 41 million people. The most frequently examined exposures were air pollution (*n* = 17), followed by noise pollution (*n* = 9), socioeconomic (*n* = 6), green and blue spaces (*n* = 3), and health and retail environments (*n* = 4). Higher concentrations of particulate matter 2.5 {PM_2.5_; hazard ratio (HR): 1.16 [95% confidence interval (95% CI): 1.09–1.24] per 10 µg/m³ increase}, higher nitrogen dioxide [NO_2_; HR: 1.05 (95% CI: 1.02–1.07) per 10 ppb increase], road traffic noise [risk ratio (RR): 1.03 (95% CI: 1.02–1.05) per 10 dB increase], and high neighbourhood-level deprivation [RR: 1.24 (95% CI: 1.17–1.31) vs. low] were each associated with increased risk of incident CVD development.

**Conclusion:**

Our findings indicate a modest yet significant increase in CVD risk associated with elevated levels of air pollution, road noise, and neighbourhood deprivation, emphasizing these exposures as consequential targets for policy intervention.

## Graphical Abstract

**Graphical Abstract:**
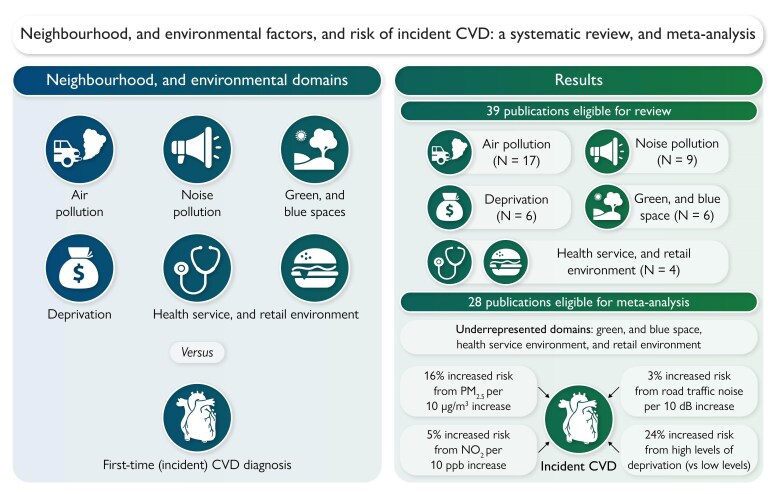

**See the editorial comment for this article ‘The heart of the matter: why cardiovascular prevention needs an environmental revolution’, by G. Guida, https://doi.org/10.1093/eurjpc/zwaf238.**

## Introduction

Social determinants of health, which encompass physical and social living conditions, are critical factors in the manifestation of cardiovascular disease (CVD). Conceptual frameworks, such as the Marmot Review, the world health organization (WHO) social determinants of health framework, and the Centres for Disease Control's Healthy People 2030 framework, categorise these determinants into key domains.^1–3^ Commonly recognized domains include socioeconomic status (SES), education, healthcare access, neighbourhood and built environment, and social context. A 2023 umbrella review of the relationship between social determinants and CVD found that most systematic reviews and meta-analyses focused on community context, with limited investigation of the impact of neighbourhood and the built environment on CVD.^4^

Where it exists, research on neighbourhood and built environment factors consistently highlights their associations with CVD mortality and morbidity.^4^ The WHO identifies air pollution as a major environmental risk factor globally, with pollutants like particulate matter (PM_2.5_) and nitrogen dioxide (NO_2_) linked to increased cardiovascular risk, particularly in areas with high vehicular emissions.^5–7^ Additionally, noise pollution—especially from road traffic—has been shown to elevate CVD risk.^8,9^ Positive environment factors, such as access to green space, are associated with a reduced risk of CVD development, while neighbourhood fast-food restaurants density correlates with increased risk.^10,11^ Moreover, both individual and neighbourhood-level SES show inverse associations with CVD development.^12^

A key limitation in the current literature is that most systematic reviews and meta-analyses focus on individual subdomains—such as pollution or green space—rather than considering multiple neighbourhood factors simultaneously.^4,13^ Furthermore, these reviews typically include a wide range of outcomes, such as hospital admissions, recurrent CVD events, and mortality, rather than focusing on CVD incidence (i.e. the first occurrence of a CVD event). Incidence is often the primary target of preventive strategies, this knowledge gap is of clinical and policy importance. Clinical prediction modelling utilizes incidence as its outcome, with models often updated in line with current and emerging evidence. Addressing knowledge gaps with reference to incidence CVD is paramount to ensuring up-to-date and relevant risk factors are included in models used by primary care physicians. Most meta-analyses concentrate solely on a specific CVD subtype, such as myocardial infarction, rather than exploring composite CVD measures, which may better reflect the overall societal burden of disease. Additionally, many reviews rely on studies with small sample sizes, increasing the potential for sampling bias. To date, no systematic reviews or meta-analyses have specifically examined the associations between neighbourhood and environmental factors and CVD incidence in large cohort studies.

Therefore, this systematic review and meta-analysis aims to investigating the relationship between neighbourhood determinants and incident CVD in large cohort studies, focusing on five key neighbourhood and environmental domains: retail environment (i.e. access to fast-food outlets, bars, and tobacconists); health services (i.e. access to primary and secondary care centres); physical environment (i.e. access to green space); pollution (air quality and noise pollution); and neighbourhood deprivation status.

## Methods

We conducted a systematic review of retrospective, prospective, and longitudinal cohort studies investigating the risk of incident CVD in people exposed to the following residential neighbourhood factors known to impact health: access to fast-food outlets, alcohol, and tobacco shops etc. (retail environment), access to primary and secondary care establishments, pharmacists etc. (health services), access to green space, urban/rural classifications etc. (physical environment) and neighbourhood-level deprivation status, and residential environmental factors: air and noise pollution (pollution).

### Search strategy

We searched four international databases for peer-reviewed articles: Ovid MEDLINE, The Cochrane Library, Web of Science, and Embase. The search strings considered a range of CVD types and neighbourhood and environmental factors known to impact health.^14^ We focussed our search using the main domains laid out in the index of ‘Access to Health Assets and Hazards’ which is an index derived of neighbourhood features known to influence health.^14^ Alternate terms, word-truncation and controlled vocabulary (i.e. Medical Subject Headings terms) were used to expand the scope of the search (see Supplementary material online, *Tables S1*–*S4*). Search strings were applied from database inception to the search date, 20th October 2024. Additional manual screening of included references and key systematic reviews were also undertaken to maximize search sensitivity.

### Study selection

All records from the database search were imported to a reference manager software (EndNote) and screened for duplicates using an automated system. EndNote reference lists were then imported into a systematic review manager software (Covidence) and an automated system removed any remaining duplicates.

One author screened article titles and abstracts for eligibility with validation by a second author. Full-text articles were then examined for eligibility, and included studies were again validated by a second author and disagreements resolved through discussion and, if needed, consulting a third author. Data extraction and quality assessment were performed and similarly confirmed by a second and third author. A data extraction table was created prior to extraction to ensure key study information was identified including sample size, population demographics, outcome and exposure measures, and any study limitations (see Supplementary material online, *Table S5*).

### Eligibility criteria

A strict eligibility criterion informed the search. Eligible studies met the following inclusion criteria: cohort studies, studies focussing on adults (≥18 years); incident CVD–composite or specific and clinically diagnosed (identification of appropriate international classification of diseases-9th/10th Revisions [ICD-9/10] codes, within electronic health records or insurance databases) as sole primary outcome [‘CVD’ included a range of coronary heart disease (CHD), cerebrovascular disease, arterial disease, atrial fibrillation (AF) (see Supplementary material online, *Tables S1*–*S4*)]; objectively measured neighbourhood factor as primary exposure, linked to patients’ area of residence; sample size of at least 1000 participants; and peer reviewed and published in English. Exclusion from the review occurred if the study met one of the following criteria: cross-sectional, case-series, or case-control study; animal studies, studies on populations with predefined systematic disease at baseline or specific demographic populations; self-reported (questionnaire or interview responses) incident CVD or studies focussing on CVD mortality (as proxy measure for incidence); subjectively measured neighbourhood factor or linked to patients’ workplace neighbourhood; and conference abstracts, oral presentations, case reports, protocols, systematic reviews, letters, and editorials. To ensure we investigated CVD incidence, studies of populations with established CVD conditions were not eligible for this review. Neighbourhood factors that were measured subjectively for example, a participant’s perception of deprivation, access to fast-food restaurants or healthcare services were excluded.

### Quality assessment

This review employed the Risk of Bias in Non-Randomized Studies of Exposures (ROBINS-E), recommended by Cochrane to determine risk of bias in cohort studies of exposures.^15^ The tool provides an approach to assess the quality of observational epidemiological studies. It is particularly helpful for assessing the quality of studies exploring exposures, such as environmental, where the use of randomized control trials is not feasible. There are seven domains where we assessed the risk of bias due to: confounding, the measurement of exposure, the selection of participants into the study (or analysis), post-exposure interventions, missing data, measurement of the outcome, and the selection of the reported results.

The review protocol was pre-registered to the PROSPERO database before the study began (registration number: CRD42023485716).

### Outcome

During data extraction, we extracted data for composite measures of CVD. When this was not possible, for example when only CVD subtypes were examined, we extracted data for the reported main outcome. We prioritized the use of data from the fully adjusted model as reported in the study because these models account for confounding. Data extracted for specific CVD types (e.g. myocardial infarction (MI), stroke, or heart failure [HF]) were termed ‘composite CVD’ for the purposes of the meta-analysis.

### Exposure

We included exposures from five specific domains: retail environment, health services, physical environment, pollution, and neighbourhood SES.

Pollution study measures were often modelled using land-use regression models and then validated against real-world monitored data; some studies used solely monitored data. The geocode of a patient’s residential address was linked to the nearest monitoring station or modelled data point. Measurements were often reported in µg/m^3^, parts per billion (ppb), and parts per million (ppm). These studies also varied in the step increases used for hazard ratios (HR), frequently using the interquartile range of measured air pollutants. To standardize the data across studies, unit conversions were performed using a common formula, applying the following standards: 10 µg/m^3^ for PM_2.5_ and PM_10_, 10ppb for NO_2_, SO_2_, and O_3_ and 1 ppm for CO. Where necessary, conversions between µg/m^3^ and ppb were also carried out.^16,17^ Since all air pollution studies reported outcomes as hazard ratios, these studies were synthesised using HR as the common measure. Similar methods were applicable to noise pollution studies, where step increases in noise pollution were standardized to 10 dB for road and rail traffic noise. Noise pollution studies reported outcomes in various formats, we converted the results to risk ratios (RR) using a standard conversion formula.^18^

Neighbourhood deprivation was measured in a range of ways, including average income level, proportion living under national poverty thresholds, and national deprivation indexes (i.e. the UK index of multiple deprivation).^19^ Most studies categorized deprivation into three levels: low, intermediate, and high. Neighbourhood deprivation studies reported outcomes in various formats, we converted the results to RR using a standard conversion formula.^18^

The levels of retail, health, or physical environment (i.e. green space) features within a set distance from the patient’s residential address was measured through satellite imagery and geographic information systems mapping.

### Statistical analyses

An evidence map was created to display all the exposure–outcome pairs examined across the included studies, displaying the studies’ reported primary and secondary outcomes.

A random-effects meta-analysis was performed to assess the risk of incident CVD across the different neighbourhood exposures. Forest plots were used to visualize the pooled results, showing the overall risk of incident CVD by each exposure. Sensitivity analyses were conducted by removing any outlier studies and those deemed to have a high risk of bias. Furthermore, there had to be at least three studies examining the same exposure to meet inclusion criteria for the meta-analysis. For studies that did not meet the eligibility criteria for inclusion in the meta-analysis, key features were narratively summarized, and descriptive statistics were included in the review.

The meta-analysis was performed in R using the ‘metafor’ package version 4.6-0.^20^ The significance level was set at *P* < 0.05.

## Results

### Study characteristics

**Figure 1:**
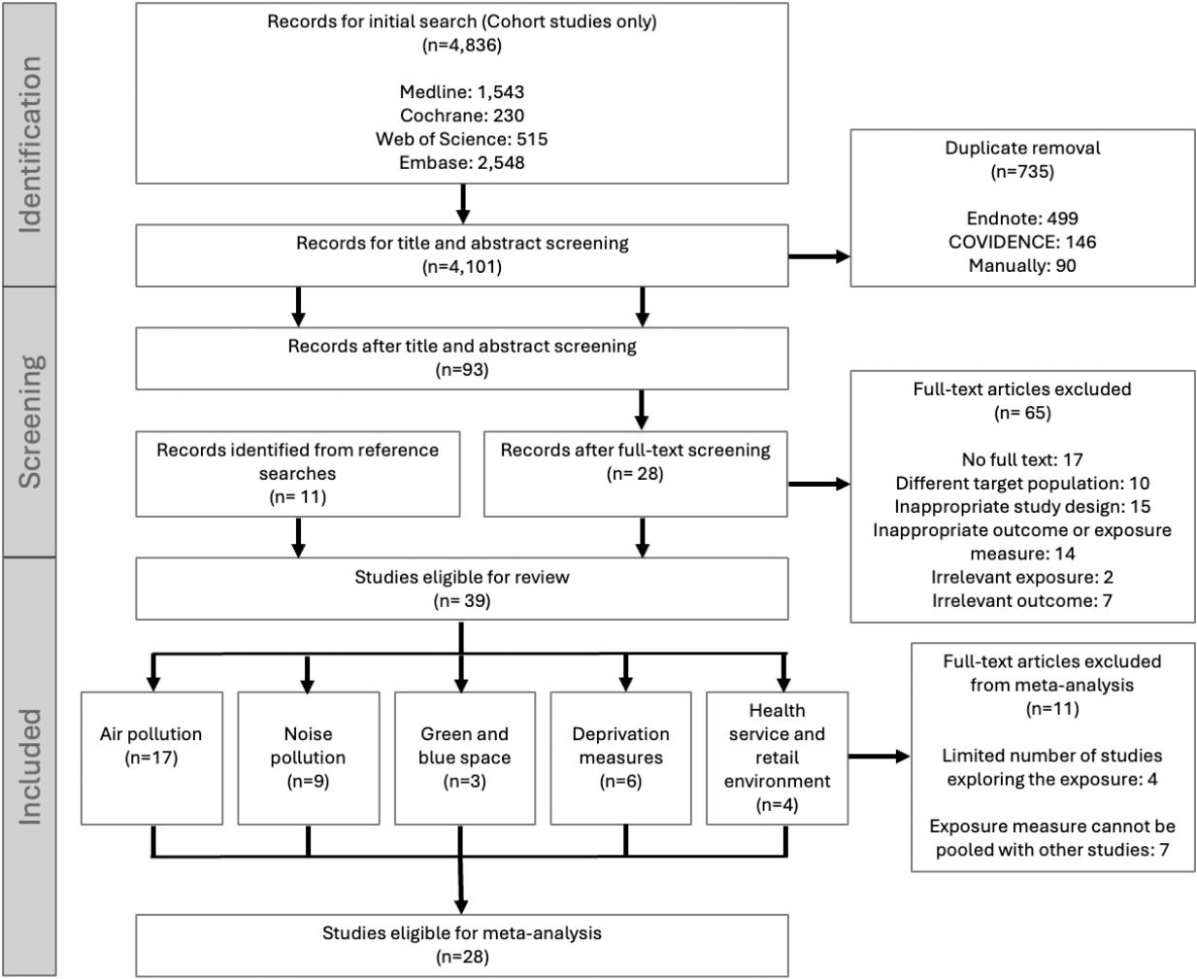
Flow chart of systematic review literature search and screening.

A total of 39 publications met the eligibility criteria and were included in the final review (flow chart, *Figure 1*). All included studies were published since 2007 (*Table 1*), with a majority (*n* = 29) being published since 2019 (*Table 1* and Supplementary material online, *Table S6*). All studies were based in either Europe (*n* = 22), North America (*n* = 9), or Asia (*n* = 8). Most studies had a sample size of over 1 million participants (*n* = 20).

**Table 1:** Characteristics of included studies

Study^a^	Continent	Age (mean)	Sex (male, %)	Sample size	Study period	Outcome(s)	Exposure(s)	ROBINS-E risk of bias
Alexeeff (2018)	North America	—	47.0	41 869	2010–15	Composite CVD^b^, MI, stroke	Air pollution	Some concerns
Atkinson (2013)	Europe	—	48.4	836 557	2003–7	MI^b^, stroke, arrhythmia, HF	Air pollution	Some concerns
Avellaneda-Gomez (2022)	Europe	47.0	48.0	3 521 274	2016–17	Isch stroke^b^	Air pollution	Some concerns
Bai (2018)	North America	51.0	47.0	1 135 817	1996–2012	HF^b^, MI	Air pollution	Some concerns
Bai (2019)	North America	53.0	48.0	5 141 172	2001–15	HF^b^, MI	Air pollution	Some concerns
Chen (2022)	Asia	44 .0	48.4	1 362 284	2010–15	Stroke^b^, Isch stroke, Haemor stroke	Air pollution	Some concerns
Gwon (2023)	Asia	57.4	46.4	292 091	2008–14	Peripheral arterial disease (PAD)^b^	Air pollution	Some concerns
Kim (2017)	Asia	42.1	49.1	136 094	2007–13	Composite CVD^b^, MI, HF, stroke, Isch stroke, Haemor stroke	Air pollution	Some concerns
Kim (2018)	Asia	—	50.1	432 587	2009–13	AF^b^	Air pollution	Some concerns
Noh (2019)	Asia	—	49.3	62 676	2002–13	Haemor stroke^b^	Air pollution	Some concerns
Olaniyan (2022)	North America	—	51.6	2 687 900	2006–16	MI^b^, stroke	Air pollution	Some concerns
Poulsen (2023)	Europe	58.0 (median)	47.0	1 964 702	2005–17	MI^b^	Air pollution	Some concerns
Shin (2019)	North America	53.2	48	5 071 956	2001–15	AF^b^, stroke	Air pollution	Some concerns
Vanoli (2024)	Europe	55.0	43.9	377 736	2006–21	Composite CVD^b^, MI, stroke, HF, AF	Air pollution	Some concerns
Wang (2021)	Europe	—	—	432 530	2006–18	HF^b^	Air pollution	Some concerns
Zhang (2019)	Asia	54.5	50.0	283 666	2000–13	Isch stroke^b^	Air pollution	Some concerns
Zhang (2024)	Europe	—	—	285 009	2006–20	Composite CVD^b^, stroke, AF, HF	Air pollution	Some concerns
Bai (2020)	North America	56.0	46.5	1 005 214	2001–15	MI^b^, HF	Noise pollution	Some concerns
Magnoni (2021)	Europe	54.0	46.4	1 087 110	2011–18	Composite CVD, MI, Isch stroke, Haemor stroke	Noise pollution	Some concerns
Poulsen (2019)	Europe	—	—	717 453	1982–2013	MI, stroke	Noise pollution	Some concerns
Poulsen (2024)	Europe	58 (median)	47	1 964 702	2005–17	MI^b^	Noise pollution	Some concerns
Seidler (2016)	Europe	—	—	854 366	2006–10	MI^b^	Noise Pollution	High risk
Sørensen (2021)	Europe	50.4	47.4	3 620 000	2000–17	Stroke^b^	Noise Pollution	High risk
Thacher (2022a)	Europe	50.4	47.8	3 604 968	2000–17	AF^b^	Noise pollution	Some concerns
Thacher (2022b)	Europe	58.6	47.5	2 538 395	2005–17	CHD^b^, MI, angina, HF	Noise pollution	Some concerns
Thacher (2024)	Europe	55.9	34.3	161 115	Up to 2017	AF^b^	Noise pollution	Some concerns
Seo (2019)	Asia	—	48.9	351 409	2006–13	Composite CVD	Green and blue space	Some concerns
Wang (2019)	North America	76.3	41.7	249 405	2010–11	MI, Isch stroke, CHD, HF, AF	Green and blue space	High risk
Xiao-Dong (2022)	Europe	55.9	45.0	377 340	2007–17	MI	Green and blue space	Low risk
Essien (2022)	North America	51.4	42.6	28 858	2005–18	AF^b^	Deprivation	Some concerns
Gwon (2020)	Asia	—	50.7	356 125	2009–13	Ischaemic heart disease (IHD)^b^	Deprivation	Some concerns
Lönn (2019)	Europe	—	47.5	3 140 657	2003–12	CHD	Deprivation	Some concerns
Pujades-Rodriquez (2014)	Europe	47.2	49.5	1 937 360	1997–2010	Composite CVD	Deprivation	Some concerns
Rethy (2021)	North America	51.4	42.6	28 858	2005–13	HF^b^	Deprivation	Some concerns
Winkleby (2007)	Europe	—	48.8	3 755 108	1996–2000	CHD^b^	Deprivation	Some concerns
Hamano (2013)	Europe	—	49.1	4 309 674	2005–7	Stroke	Food and healthcare environment	High risk
Kawakami (2011)	Europe	—	49.2	2 165 000	2005–7	CHD	Food and healthcare environment	Some concerns
Pinho (2024)	Europe	54.6	48.7	4 641 435	2004–18	Composite CVD, CHD, stroke, HF	Food and healthcare environment	Some concerns
Poelman (2020)	Europe	59.3	46.0	2 472 004	2009–10	Composite CVD, CHD, stroke, HF	Food and healthcare environment	Some concerns

^a^Study references can be found in Supplementary material online, *Table S6*.

^b^Primary outcome used for composite CVD measure in the meta-analysis.

**Figure 2:**
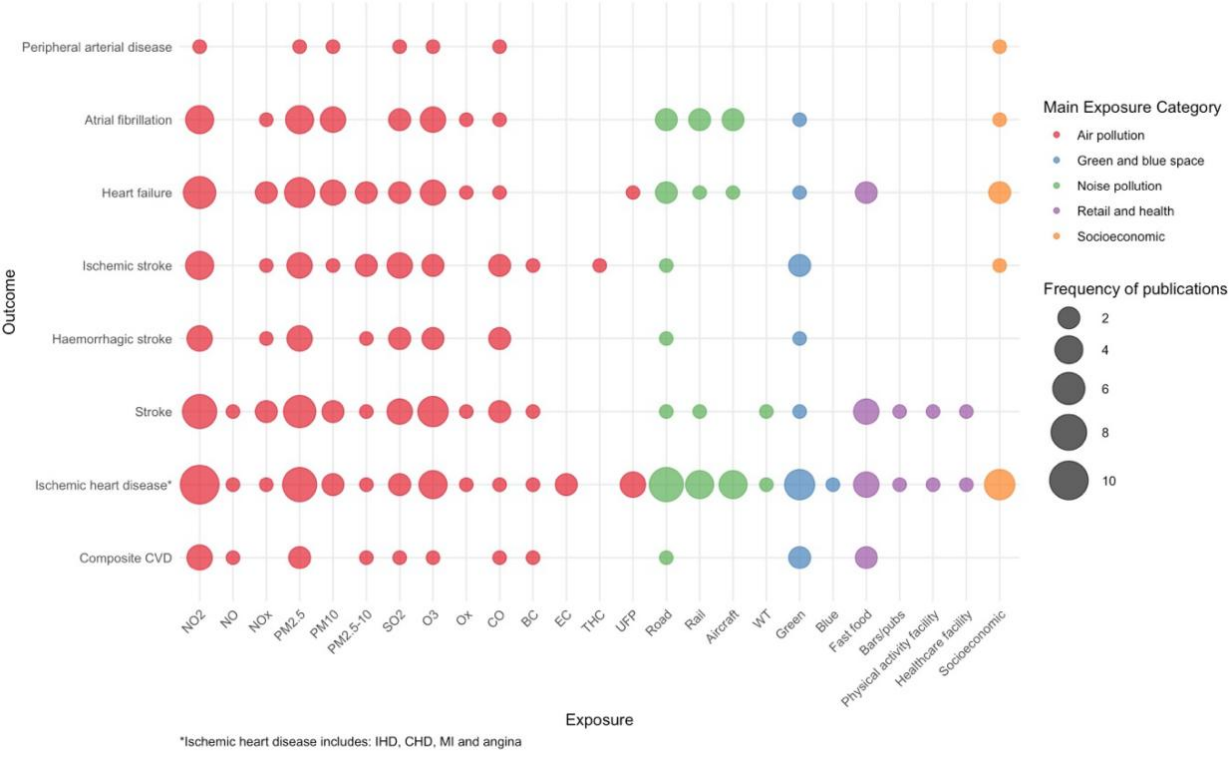
Evidence map of all included studies.

Most of the included studies examined air pollution as the main exposure (*n* = 17), followed by noise pollution (*n* = 9), measures of deprivation, socioeconomic or poverty (*n* = 6), retail environment (*n* = 4), and green and blue space (*n* = 3) (*Table 1*). The most studied primary outcomes for CVD incidence were myocardial infarction (*n* = 12), composite CVD measure (*n* = 8), and stroke (*n* = 6) (*Table 1*). The evidence map shows the range of outcomes and exposures examined across all included studies (*n* = 39) (*Figure 2*). Ischaemic heart disease was the most frequent outcome across most exposure domains, with air pollution the most frequently explored exposure. Evidence was lacking in studies examining associations between stroke, atrial fibrillation and heart failure and noise pollution, green and blue space, the health and retail environment and neighbourhood-level deprivation.

We excluded 11 publications from the meta-analysis due to either a lack of studies examining the same exposure or an inability to extract appropriate outcome data, leaving an analytical cohort of 28 publications representing over 41.8 million people (*Figure 1*).

### Risk of bias

There were 34 studies categorized as having some concern of risk of bias, four studies were classified as having a high risk of bias, and one study was assessed to have a low risk of bias (*Table 1* and Supplementary material online, *Table S7*). Studies not adequately accounting for potential confounding and missing data were the main contributors to higher risk of bias. For pollution studies, there was a risk of publication bias as demonstrated using NO_2_ as an exposure by the asymmetrical arrangement of studies around our estimated outcome in the funnel plot and through a statistically significant *P*-value (*P* < 0.01) from the Egger’s test for funnel plot asymmetry (see Supplementary material online, *Figure S1*).

### Association between exposures and incident cardiovascular disease

#### Air pollution

**Figure 3:**
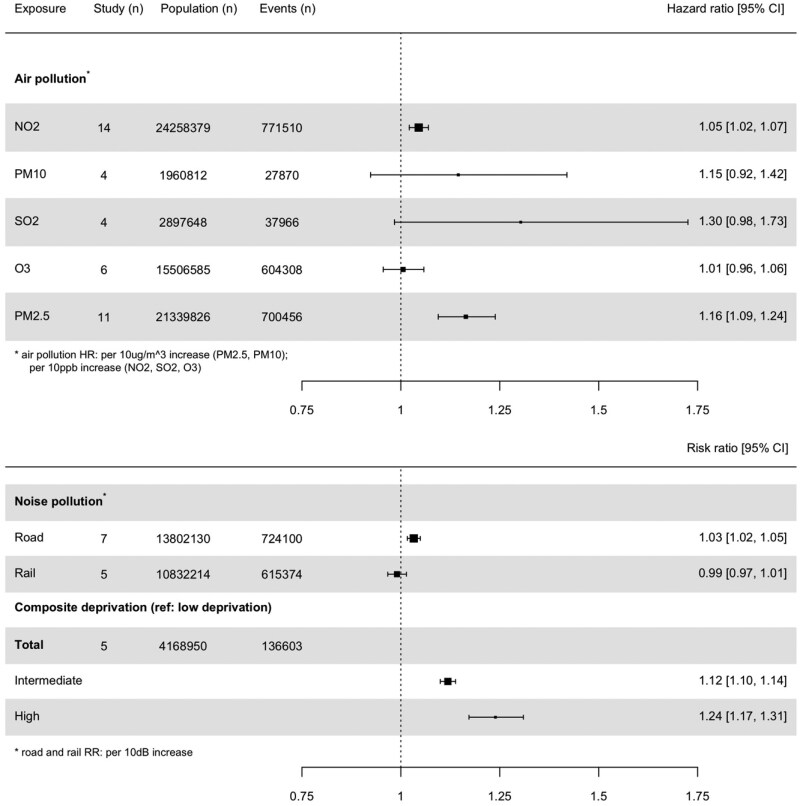
Pooled hazard and risk ratios across three exposure domains.

The most frequently explored air pollutants across included studies are NO_2_ (*n* = 16), PM_2.5_ (*n* = 15), and O_3_ (*n* = 7). Air pollutants meeting the criteria for inclusion in the random-effects meta-analysis (PM_2.5_, PM_10_, NO_2_, SO_2_, O_3_) showed a significant association between higher concentrations of NO_2_ in residential areas and an increased risk of incident CVD [HR = 1.05 per 10 ppb increase, 95% confidence interval (95% CI): 1.02–1.07] (*Figure 3* and Supplementary material online, *Figure S2*). Similarly, exposure to PM_2.5_ was associated with a higher risk of incident CVD (HR = 1.16 per 10 µg/m^3^ increase, 95% CI: 1.09–1.24) (*Figure 3* and Supplementary material online, *Figure S3*).

For other pollutants, such as PM_10_ and SO_2_, the pooled hazard ratios indicated a potential increase in the risk of incident CVD; however, these associations were not statistically significant in the context of wide CIs (*Figure 3* and Supplementary material online, *Figures S4*–*S6*). Additionally, no significant association was observed between ozone (O_3_) and the risk of incident CVD (HR = 1.01 per 10ppb increase, 95% CI: 0.96–1.06).

#### Noise pollution

A total of nine studies investigated the relationship between noise pollution and the risk of incident CVD, examining various noise sources, including road traffic noise (*n* = 8), railway noise (*n* = 5), aircraft noise (*n* = 4), and wind-turbine noise (*n* = 1).

In the random-effects meta-analysis, exposure to higher levels of road traffic noise, compared with lower levels of road traffic noise, was found to be significantly associated with an increased risk of incident CVD (RR = 1.03, 95% CI: 1.02–1.05) (*Figure 3* and Supplementary material online, *Figure S7*). However, when pooling estimates for railway noise exposure, no significant association with incident CVD risk was observed (*Figure 3* and Supplementary material online, *Figure S8*).

#### Neighbourhood deprivation

Six studies examined the relationship between neighbourhood-level deprivation and the risk of incident CVD. In the meta-analysis, strong positive associations were found between neighbourhood deprivation and the risk of incident CVD. Specifically, individuals living in areas with intermediate levels of deprivation (RR = 1.12, 95% CI: 1.10–1.14) and high deprivation (RR = 1.24, 95% CI: 1.17–1.31) showed sequentially higher risk of incident CVD compared with individuals living in low-deprivation neighbourhoods (*Figure 3* and Supplementary material online, *Figures S9* and *S10*).

#### Green and blue space

Two studies examined the relationship between access to green space and the risk of incident CVD. One study measured exposure by accessing the percentage of green space within the local area, while the other used the normalized difference vegetation index as the exposure measure. Both studies individually reported that greater access to green space was associated with a reduced risk of incident CVD.

#### Retail and health environment

Two studies explored the associations between CVD incidence and access to retail environments (fast-food outlets, bars, and pubs) and health-related facilities (physical activity centres and healthcare facility). Additionally, one study specifically examined the effect of fast-food outlets access on CVD risk, and another study assessed the food environment more generally utilizing a food environment index. Among the four studies that explored fast-food outlet access, three found that greater access was associated with in increased incidence of stroke. However, no significant associations were found between access to physical activity facilities or healthcare services and the risk of incident CVD.

#### Sensitivity and subgroup analysis

One study on air pollution had been identified as an extreme outlier from a funnel plot (see Supplementary material online, *Figure S11*).^21^ It was decided to exclude this study from the main analysis, we have included the pooled analysis with this study included in the Supplementary material online, material (see Supplementary material online, *Figure S12*). Sensitivity analysis, performed by excluding studies classified as high risk of bias using the ROBINS-E tool, showed minimal effect on the overall results (see Supplementary material online, *Figure S13*).

Due to the number of studies available, subgroup analysis focused primarily on air pollution exposures. Pooled estimates for NO_2_ were consistent across different regions, except for studies from Asia, where no significant association between NO_2_ exposure and CVD incidence was observed (see Supplementary material online, *Figure S14*). The association between PM_2.5_ and CVD incidence was stronger in studies from Asia and Europe. However, the pooled estimate for European studies had a wide CI, indicating greater uncertainty in these findings.

Studies published from 2020 onward reported stronger associations between both NO_2_ and PM_2.5_ and CVD incidence (see Supplementary material online, *Figure S15*). Additionally, studies with a sample size over 1 million participants demonstrated significant associations between PM_2.5_ and NO_2_ exposure and CVD incidence, with narrower CIs, suggesting higher precision in the estimate (see Supplementary material online, *Figure S16*). In contrast, studies with under 1 million participants also reported associations between PM_2.5_ and CVD incidence, but the CIs were much wider, indicating less precision. No significant association was found between NO_2_ exposure and CVD incidence in these smaller studies.

## Discussion

In this systematic review and meta-analysis of the impact of neighbourhood and environmental factors on incident CVD, including data from over 41.8 million participants, we found statistically significant associations of increased risk of incident CVD with higher concentrations of PM_2.5_ and NO_2_, increased road traffic noise, and neighbourhood deprivation. There was limited reported research on the effects of access to green or blue spaces, as well as the health and retail environment on incident CVD, and a notable lack of geographic diversity, particularly for low- and middle-income countries.

This study contributes novel insights to the literature by focusing exclusively on incident CVD, an outcome that has frequently been overlooked in previous reviews, which largely examined the associations between neighbourhood factors and CVD mortality or recurrent events.^5,8,22^ This focus is important because incident CVD is a key target for preventive strategies and developing risk prediction models. We only included large cohort studies with objective measures of neighbourhood exposures therefore our meta-analysis minimises biases that are more prevalent in smaller studies or those reliant on self-reported data, thus providing more reliable risk estimates. Systematic reviews that included many more cross-sectional studies were likely to report more studies with a greater risk of bias and report greater outcome–exposure associations.^23^ Our systematic review included a minimal number of studies with a high risk of bias, and our results align with others in the subject area.^5,24^

Neighbourhood factors can directly influence clinical and behavioural risk factors, and they can interact with each other to either amplify or mitigate these effects. Air pollution, particularly pollutants from exhaust and other combustion sources like PM_2.5_ and NO_2_, when inhaled, directly increases CVD risk, through contributing to inflammation, oxidative stress, and endothelial dysfunction.^25–27^ Noise pollution, through mechanisms such as sleep disruption and increased stress, has similarly been linked to adverse cardiovascular outcomes.^9,28^ On the other hand, access to green space promotes physical activity and an active lifestyle, while also reducing stress, which benefits cardiovascular health.^11^

Environmental exposures often coexist, particularly in socioeconomically deprived areas, compounding the overall risk of CVD. Areas with higher poverty levels typically experience more air and noise pollution, as they are often located near highways and industrial centres. These areas also tend to have less access to green space, limiting opportunities for physical activity and removing natural buffers against pollution.^29^ These synergistic effects between neighbourhood and environmental exposures and neighbourhood deprivation may explain why we observed the highest magnitude of association between the risk of CVD development and greater levels of neighbourhood deprivation compared to any single measure of other domains such as NO_2_. Use of an exposome approach, which examines the cumulative risk associated with interactions between several environmental exposures, clinical factors, and epigenetic variations over time, may be implemented by researchers in future studies to help examine these emerging risk factors for CVD.^30,31^

Our findings emphasize the need to implement strategies to mitigate the impact of noise pollution, air pollutants and neighbourhood deprivation on incident CVD. The WHO and The European Society of Cardiology have advocated national governments to lower noise exposure.^32–34^ Despite 20% of urban populations across Europe being exposed to levels above 55 dB, which is above the WHO-recommended levels, governments do not often make specific targets to reduce the health burden from noise pollution.^32^ National governments should seek to rectify this and seek to set targets for reductions in noise exposure. Improving traffic management has the joint benefit of both reducing traffic-borne air pollution and reducing road traffic noise. There is consistent evidence that the introduction of low emission zones reduces adverse cardiovascular outcomes.^35^ Higher levels of deprivation often exist alongside higher levels of pollution and noise exposure, and reduced access to green space. A report from the UK found that green areas in neighbourhoods with high levels of deprivation are often built upon.^36^ They suggest a need for more designated green spaces, protected in law, which are often lacking in areas of high deprivation. Combinations of these policy decisions targeting different neighbourhood domains could enhance the positive effects on cardiovascular events.

We also identified important gaps in the research, particularly regarding the influence of green and blue spaces, access to health services, and the retail environment on CVD incidence. Researchers may want to examine urban and rural distinctions within these underrepresented domains, research suggests rural communities are at a higher risk of mortality following myocardial infarction in part due to differences in health service access.^37^ Our review showed an absence of large cohort studies exploring associations between environmental factors in CVD incidence in the global south. Populations in developing countries are more likely to be exposed to greater levels of both indoor and outdoor air pollution.^38^ Indoor pollution and extreme levels of outdoor pollution are likely to be more impactful on certain regions. Soon, extreme heat and weather fluctuations may become more prominent environmental risk factors, particularly for regions around the world most affected by climate change.^39^ It is important to expand the evidence base on environmental risk factors to show and compare region specific risk factors for CVD. Although, we should be mindful during the research process to not infer causality on highly complex interactions. We found several neighbourhood-level factors associated with incident CVD. Future research could examine how incorporating neighbourhood-level factors into CVD risk modelling to improve risk prediction. This could lead to reclassifying patients into higher risk categories, something often under predicted in current prediction models, enabling earlier interventions, that is.^40^

The lack of geographic diversity in the studies included in our meta-analysis, with the majority originating from high-income countries, limits the generalizability of our findings to low- and middle-income countries where the burden of CVD and environmental risk factors may differ significantly. Researchers should advocate for funding to be allocated to help create and maintain public datasets in low- and middle-income countries and additionally expand digital infrastructure and training which could help facilitate storage and access to retrospective health data archives.^41,42^ Improvements like these can help facilitate health research in these geographic areas that are underrepresented in research on emerging risk factors for CVD. The focus of our paper on neighbourhood-level exposure studies that used large population-based cohort studies may have excluded emerging pollutants that are more likely to be studied using smaller sample sizes at the individual level. We restricted our search to studies written in English, though this has not been found to lead to significant bias.^43^ We limited our review to exposure measures taken at patients’ residential addresses. We acknowledge that measures taken at a fixed location can introduce bias by overlooking the significant amount of time patients spend elsewhere, patients may move residences, and important spatiotemporal contexts in the levels of exposure for any individual may be absent.^44^ We attempted to minimize measurement bias by only including studies that used objectively measured exposures, such as air pollution, and these often-used small area measurements.

## Conclusion

This systematic review, including data from over 41 million participants, highlights the role of neighbourhood factors—air pollution, noise pollution, and deprivation—in increasing risk of the development of CVD. Strategies like improved traffic management, expanding green spaces, and enhancing the built environment in deprived areas can reduce CVD risk and address health inequalities. Incorporating environmental factors into CVD risk models could also enable more targeted preventive measures, ultimately helping to lower the global CVD burden.

## Supplementary Material

zwaf165_Supplementary_Data

## Data Availability

All data used in the study are publicly available and can be made available upon request.
