# Supplementary material for: Impact of neighbourhood and environmental factors on the risk of incident cardiovascular disease: a systematic review and meta-analysis
Source: Eur J Prev Cardiol. 2025 Mar 19;32(18):1903–12. doi: 10.1093/eurjpc/zwaf165 (PMC12721861; doi:10.1093/eurjpc/zwaf165)
Supplement: zwaf165_Supplementary_Data [file zwaf165_supplementary_data.docx]

**Title:** Impact of neighbourhood and environmental factors on the risk of incident cardiovascular disease: a systematic review and meta-analysis

**Author:** Jack R.G. Brown^a^, Paris Baptiste^a^, Hajar Hajmohammadi^a^, Ramesh Nadarajah^b,c,d^, Chris P. Gale^b,c,d^, Jianhua Wu^a^

^a^Centre for Primary Care, Wolfson Institute of Population Health, Queen Mary University of London, London, UK

^b^Leeds Institute of Data Analytics, University of Leeds, Leeds, UK

^c^Leeds Institute for Cardiovascular and Metabolic Medicine, University of Leeds, Leeds, UK

^d^Department of Cardiology, Leeds Teaching Hospitals NHS Trust, Leeds, UK

**Supplemental material**

**Table S1: MEDLINE (ovid) search string (****up to 20/10/2024)**

| **#** | **Query** | **Results** |
| --- | --- | --- |
| 1 | (“atherosclerosis” OR “cerebral infarction” OR “cardiovascular disease*” OR “cerebrovascular disorder*” OR Hypertension OR “Isch?emi* attack” OR “myocardial isch?emi*” OR “myocardial infarction” OR “peripheral vascular disease*” OR “peripheral arterial disease*” OR “vascular disease*” OR “stroke” OR ((cardiovascular OR cardiac) adj2 (mortality OR death OR sudden OR adverse)) OR (Atrial adj (fibrillation OR flutter)) OR (Angina adj (pectoris OR stable OR unstable)) OR (Carotid adj2 disease*) OR (Coronary adj2 (disease* OR syndrome)) OR (Heart adj (arrest* OR attack* OR disease* OR failure*))).ti. | 820,178 |
| 2 | cardiovascular diseases/ | 188,019 |
| 3 | death, sudden, cardiac/ | 18,776 |
| 4 | heart diseases/ | 75,853 |
| 5 | vascular diseases/ | 40,031 |
| 6 | cerebrovascular disorders/ | 48,340 |
| 7 | stroke/ | 142,197 |
| 8 | myocardial infarction/ | 183,810 |
| 9 | 1 OR 2 OR 3 OR 4 OR 5 OR 6 OR 7 OR 8 | 1,201,756 |
| 10 | Incidence/ or inciden*.ab,ti. | 1,268,840 |
| 11 | Prevalence/ or prevalen*.ab,ti | 1,154,680 |
| 12 | Risk/ or Risk Factors/ or risk*.ab,ti. | 3,529,458 |
| 13 | Disease management/ or medication therapy management/ or case management/ or therapeutics/ or (management or treatment or therap*).ti. | 2,790,121 |
| 14 | (10 OR 11 OR 12) NOT 13 | 4,502,671 |
| 15 | Cohort Studies/ or (cohort adj (stud* or analy*)).ab,ti. or longitudinal.ab,ti. or (prospective adj2 (stud* or analy* or data)).ab,ti. or (retrospective adj2 (stud* or analy* or data)).ab,ti. | 1,627,444 |
| 16 | Cross-sectional studies/ or (cross-sectional adj (study* or analy* or data)).ab,ti. or Randomized control trials as topic/ or “randomi*ed control trial*”.ab,ti. or case-control studies/ or (case adj control adj2 (stud* or analy* or data)).ab,ti. | 997,316 |
| 17 | Systematic Review/ or Meta-Analysis/ or Letter/ or editorial/ or comment/ or clinical trial protocol/ or “systematic review*”.ti. or “meta-analys*”.ti. or (meta adj analys*).ti. or (letter or comment or editorial or protocol).ti. | 2,880,708 |
| 18 | Infant/ or Child/ or Adolescent/ or (adolescen* or child* or schoolchild* or infant* or girl* or boy* or teen* or youth* or p?ediatr* or puber*).ab,ti. | 4,544,625 |
| 19 | 15 NOT (16 OR 17 OR 18) | 1,057,974 |
| 20 | Residence Characteristics/ or Population Density/ or Cities/ or Home Environment/ or Environment Design/ or Built Environment/ | 100,668 |
| 21 | ((Neighbo?rhood* or communit* or residence* or built* or contextual* or geograph* or urban or rural or environment* OR cit* or home) adj2 (determinant* or factor* or environment* or characteristic* or exposure or attribute* or area or region or impact or effect or design)).ti. | 240,094 |
| 22 | Health Facility Environment/ or Health Facility Planning/ or Medically Underserved Area/ | 13,865 |
| 23 | ((“health service*" or “health care” or care or GP* or "general practice*" or hospital* or “secondary care” or “primary care” or pharmacy or pharmacist or dentist or “leisure service*” or "leisure cent*" or “recreational cent*”) adj2 (access or “access to” or quality or “quality of” or inaccess* or lack* or “lack of” or environment* or disparit*)).ab,ti. | 141,409 |
| 24 | Social Determinants of Health/ or Sociological Factors/ or Socioeconomic Factors/ or Socioeconomic Disparities in Health/ or Poverty/ or Poverty Areas/ or Social Deprivation/ | 225,217 |
| 25 | (“social determinant*” or socio?economic* or disparit* or poverty or deprivation or segregation* or insecurity or security).ti. | 117,652 |
| 26 | Environmental Pollution/ or Air Pollution/ or Traffic-Related Pollution/ or Water Pollution/ or Light Pollution/ or Noise, Transportation/ or Parks, recreational/ or walking/ or transportation/ | 136,733 |
| 27 | (pollut* or “air quality”).ti. | 65,245 |
| 28 | (“green space*” or “blue space*” or walkability or transport* or transit).ab,ti. | 703,661 |
| 29 | Food Deprivation/ or Food Supply/ or nutrition value/ | 41,347 |
| 30 | (("fast?food*" or food* or retail* or grocery* or supermarket* or shop* or nutrition* or convenience* or restaurant* or nightlife* or pub* or bar* or nightclub* or off?license* OR "liquor store*" or tobacconist* or "gambling outlet*") adj2 (access or “access to” or quality or “quality of” or inaccess* or lack* or “lack of” or environment* or disparit*)).ab,ti. | 66,341 |
| 31 | 20 OR 21 OR 22 OR 23 OR 24 OR 25 OR 26 OR 27 OR 28 OR 29 OR 30 | 1,655,041 |
| 32 | (national or nationwide or registry or countrywide or community or population or electronic health record*).ab,ti,kw. or electronic health records/ | 3,160,530 |
| 33 | 9 AND 14 AND 19 AND 31 AND 32 | 1,574 |
| 34 | Limit 33 to English | 1,543 |

**Table S2: Cochrane library search string (up to 20/10/2024)**

| **#** | **Query** | **Results** |
| --- | --- | --- |
| 1 | atherosclerosis:ti OR cerebral infarction:ti OR cardiovascular disease*:ti OR cerebrovascular disorder*:ti OR hypertension:ti OR isch?emi* attack:ti OR myocardial isch?emi*:ti OR myocardial infarction:ti OR peripheral vascular disease*:ti OR peripheral arterial disease*:ti OR vascular disease*:ti OR stroke:ti OR (((cardiovascular OR cardiac) NEAR/2 (mortality OR death OR sudden OR adverse)):ti) OR ((atrial NEXT/1 (fibrillation OR flutter)):ti) OR ((angina NEXT/1 (pectoris OR stable OR unstable)):ti) OR (carotid NEXT/2 disease*):ti OR ((coronary NEXT/2 (disease* OR syndrome)):ti) OR ((heart NEXT/1 (arrest* OR attack* OR disease* OR failure*)):ti) | 142,594 |
| 2 | [mh ”cardiovascular diseases”] | 157,958 |
| 3 | [mh ”death, sudden, cardiac”] | 947 |
| 4 | [mh ”heart diseases”] | 75,149 |
| 5 | [mh ”vascular diseases”] | 117,302 |
| 6 | [mh ”cerebrovascular disorders”] | 25,728 |
| 7 | [mh stroke] | 17,885 |
| 8 | [mh ”myocardial infarction”] | 15,784 |
| 9 | #1 OR #2 OR #3 OR #4 OR #5 OR #6 OR #7 OR #8 | 232,981 |
| 10 | [mh Incidence] OR inciden*:ti,ab | 167,701 |
| 11 | [mh Prevalence] OR prevalen*:ti,ab | 59,070 |
| 12 | [mh Risk] OR [mh ”Risk Factors”] OR risk*:ti,ab | 311,630 |
| 13 | [mh ”Disease management”] OR [mh ”medication therapy management”] OR [mh ”case management”] OR [mh therapeutics] OR (management OR treatment OR therapeutics):ti | 680,626 |
| 14 | (#10 OR #11 OR #12) NOT #13 | 312,248 |
| 15 | [mh ”Cohort Studies”] OR (cohort NEXT/1 (stud* or analy*)):ti,ab OR longitudinal:ti,ab OR (prospective NEXT/2 (stud* OR analy* OR data)):ti,ab OR (retrospective NEXT/2 (stud* or analy* or data)):ti,ab | 278,335 |
| 16 | [mh ”Cross-sectional studies”] OR (cross-sectional NEXT/2 (study* OR analy* OR data)):ti,ab OR [mh ”Randomized control trials as topic”] OR randomi*ed control trial*:ti,ab OR [mh ”case-control studies”] OR (case NEXT/1 control NEXT/2 (stud* OR analy* OR data)):ti,ab | 863,185 |
| 17 | [mh ”Systematic Review”] OR [mh ”Meta-Analysis”] OR [mh Letter] OR [mh editorial] OR [mh comment] OR [mh ”clinical trial protocol”] OR systematic review*:ti OR meta-analys*:ti OR (meta NEXT/1 analys*):ti OR (letter OR comment OR editorial OR protocol):ti | 47,347 |
| 18 | [mh Infant] OR [mh Child] OR [mh Adolescent] OR (adolescen* OR child* OR schoolchild* OR infant* OR girl* OR boy* OR teen* OR youth* OR p?ediatr* OR puber*):ti,ab | 357,936 |
| 19 | #15 NOT (#16 OR #17 OR #18) | 99,062 |
| 20 | [mh ”Residence Characteristics”] OR [mh ”Population Density”] OR [mh Cities] OR [mh ”Home Environment”] OR [mh ”Environment Design”] OR [mh ”Built Environment”] | 6,845 |
| 21 | ((Neighbo?rhood* OR communit* OR residence* OR built* OR contextual* OR geograph* OR urban OR rural OR environment* OR cit* or home) NEAR/2 (determinant* OR factor* OR environment* OR characteristic* OR exposure OR attribute* OR area OR region OR impact OR effect or design)):ti | 4,989 |
| 22 | [mh ”Health Facility Environment”] OR [mh ”Health Facility Planning”] OR [mh ”Medically Underserved Area”] | 340 |
| 23 | ((health service* OR health care OR care OR GP* OR general practice* OR hospital* OR secondary care OR primary care OR pharmacy OR pharmacist OR dentist OR leisure service* OR leisure cent* OR recreational cent*) NEAR/2 (access OR access to OR quality OR quality of OR inaccess* OR lack* OR lack of OR environment* OR disparit*)):ti,ab | 474,514 |
| 24 | [mh ”Social Determinants of Health”] OR [mh ”Sociological Factors”] OR [mh ”Socioeconomic Factors”] OR [mh ”Socioeconomic Disparities in Health”] OR [mh Poverty] OR [mh ”Poverty Areas”] OR [mh ”Social Deprivation”] | 31,397 |
| 25 | (social determinant* OR socio?economic* OR disparit* OR poverty OR deprivation OR segregation* OR insecurity OR security):ti | 4,697 |
| 26 | [mh ”Environmental Pollution”] OR [mh ”Air Pollution”] OR [mh ”Traffic-Related Pollution”] OR [mh ”Water Pollution”] OR [mh ”Light Pollution”] OR [mh ”Noise, Transportation”] OR [mh ”Parks, recreational”] OR [mh walking] OR [mh transportation] | 14,928 |
| 27 | (pollut* OR air quality):ti | 671 |
| 28 | (green space* OR blue space* OR walkability OR transport* OR transit):ti,ab | 9,186 |
| 29 | [mh ”Food Deprivation”] OR [mh ”Food Supply”] OR [mh ”nutrition value”] | 2,168 |
| 30 | ((fast?food* OR food* OR retail* OR grocery* OR supermarket* OR shop* OR nutrition* OR convenience* OR restaurant* OR nightlife* OR pub* OR bar* OR nightclub* OR off?license* OR liquor store* OR tobacconist* OR gambling outlet*) NEAR/2 (access OR access to OR quality OR quality of OR inaccess* OR lack* OR lack of OR environment* OR disparit*)):ti,ab | 81,668 |
| 31 | #20 OR #21 OR #22 OR #23 OR #24 OR #25 OR #26 OR #27 OR #28 OR #29 OR #30 | 569,374 |
| 32 | (national OR nationwide OR registry OR countrywide OR community OR population OR electronic health record*):ti,ab,kw OR [mh ”electronic health records”] | 269,074 |
| 33 | #9 AND #14 AND #19 AND #31 AND #32 | 236 |
| 34 | #33 AND English:la | 230 |

**Table S3: Web of science search string (up to 20/10/2024)**

| **#** | **Query** | **Results** |
| --- | --- | --- |
| 1 | TI=(Atherosclerosis OR cerebral infarction OR cardiovascular disease* OR cerebrovascular disorder* OR cerebrovascular disease* OR hypertension OR isch?emi* attack OR myocardial isch?emi* OR myocardial infarction OR peripheral vascular disease* OR peripheral arterial disease* OR vascular disease* OR stroke OR (atrial NEXT/1 (fibrillation OR flutter)) OR (angina NEXT/1 (pectoris OR stable OR unstable)) OR (carotid NEXT/2 disease*) OR (coronary NEXT/2 (disease* OR syndrome)) OR (heart NEXT/1 (arrest* OR attack* OR disease* OR failure*))) | 690,408 |
| 2 | TI=(((cardiovascular OR cardiac) NEAR/2 (mortality OR death OR sudden OR adverse))) | 30,378 |
| 3 | TS=(cardiovascular disease*) | 506,241 |
| 4 | #1 OR #2 OR #3 | 1,093,516 |
| 5 | TS=(inciden*) | 1,398,538 |
| 6 | TS=(prevalen*) | 1,564,304 |
| 7 | TS=(risk or risk factor*) | 4,707,738 |
| 8 | TI=(management OR treatment OR therap*) | 3,601,036 |
| 9 | (#5 OR #6 OR #7) NOT #8 | 6,005,899 |
| 10 | TS=((cohort NEXT/1 (stud* or analy*)) OR longitudinal OR (prospective NEXT/2 (stud* OR analy* OR data)) OR (retrospective NEXT/2 (stud* or analy* or data))) | 621,108 |
| 11 | TS=((cross-sectional NEXT/2 (study* OR analy* OR data)) OR randomi*ed control trial* OR (case NEXT/1 control NEXT/2 (stud* OR analy* OR data))) | 581,042 |
| 12 | TI=(systematic review* OR meta-analys* OR (meta NEXT/1 analys*) OR (letter OR comment OR editorial OR protocol)) | 1,063,074 |
| 13 | TS=(adolescen* OR child* OR schoolchild* OR infant* OR girl* OR boy* OR teen* OR youth* OR p?ediatr* OR puber*) | 3,390,377 |
| 14 | #10 NOT (#11 OR #12 OR #13) | 487,586 |
| 15 | TS=("Residence Characteristics" OR "Population Density" OR "Cities" OR "Home Environment" OR "Environment Design" OR "Built Environment") | 344,700 |
| 16 | TI=(("Neighbo?rhood"* OR "communit*" OR "residence*" OR "built*" OR "contextual*" OR "geograph*" OR "urban" OR "rural" OR "cit*" OR "environment*" OR "home") NEAR/2 ("determinant*" OR "factor*" OR "environment*" OR "characteristic*" OR "exposure" OR "attribute*" OR "area" OR "region" OR "impact" OR "effect" OR "design")) | 828,690 |
| 17 | TS=(“Health Facility Environment” OR “Health Facility Planning” OR “Medically Underserved Area”) | 418 |
| 18 | TS=((“health service*” OR “health care” OR “care” OR “GP” OR “general practice*” OR “hospital*” OR “secondary care” OR “primary care” OR “pharmacy” OR “pharmacist” OR “dentist” OR “leisure service*” OR “leisure cent*” OR “recreational cent*”) NEAR/2 (“access” OR “access to” OR “quality” OR “quality of” OR “inaccess*” OR “lack*” OR “lack of” OR “environment*” OR “disparit*”)) | 216,152 |
| 19 | TS=(“Social Determinants of Health” OR “Sociological Factors” OR “Socioeconomic Factors” OR “Socioeconomic Disparities in Health” OR “Poverty” OR “Poverty Areas” OR “Social Deprivation”) | 159,090 |
| 20 | TI=(“social determinant*” OR “socio?economic*” OR “disparit*” OR “poverty” OR “deprivation” OR “segregation*” OR “insecurity” OR “security”) | 297,746 |
| 21 | TS=(“Environmental Pollution” OR “Air Pollution” OR “Traffic-Related Pollution” OR “Water Pollution” OR “Light Pollution” OR “Noise Transportation” OR “Parks recreational” OR “walking” OR “transportation”) | 624,951 |
| 22 | TI=(“pollut*” OR “air quality”) | 163,296 |
| 23 | TS=(“green space*” OR “blue space*” OR “walkability” OR “transport*” OR “transit”) | 2,171,291 |
| 24 | TS=(“Food Deprivation” OR “Food Supply” OR “nutrition value”) | 25,444 |
| 25 | TS=((“fast?food*” OR “food*” OR “retail*” OR “grocery*” OR “supermarket*” OR “shop*” OR “nutrition*” OR “convenience*” OR “restaurant*” OR “nightlife*” OR “pub*” OR “bar*” OR “nightclub*” OR “off?license*” OR “liquor store*” OR “tobacconist*” OR “gambling outlet*”) NEAR/2 (“access” OR “access to” OR “quality” OR “quality of” OR “inaccess*” OR “lack*” OR “lack of” OR “environment*” OR “disparit*”)) | 228,580 |
| 26 | **#25 OR #24 OR #23 OR #22 OR #21 OR #20 OR #19 OR #18 OR #17 OR #16 OR #15** | 4,378,792 |
| 27 | TS=(national OR nationwide OR registry OR countrywide OR community OR population OR electronic health record*) | 6,266,993 |
| 28 | #4 AND #9 AND #14 AND #26 AND #27 | 522 |
| 29 | **#4 AND #9 AND #14 AND #26 AND #27** and **English** (Languages) | 515 |

**Table S4: EMBASE search string (up to 20/10/2024)**

| **#** | **Query** | **Results** |
| --- | --- | --- |
| 1 | 'atherosclerosis':ti OR 'cerebral infarction':ti OR 'cardiovascular disease*':ti OR 'cerebrovascular disorder*':ti OR 'hypertension':ti OR 'isch?emi* attack':ti OR 'myocardial isch?emi*':ti OR 'myocardial infarction':ti OR 'peripheral vascular disease*':ti OR 'peripheral arterial disease*':ti OR 'vascular disease*':ti OR 'stroke':ti OR ((('cardiovascular' OR 'cardiac') NEAR/2 ('mortality' OR 'death' OR 'sudden' OR 'adverse')):ti) OR (('atrial' NEXT/1 ('fibrillation' OR 'flutter')):ti) OR (('angina' NEXT/1 ('pectoris' OR 'stable' OR 'unstable')):ti) OR ('carotid' NEXT/2 'disease*'):ti OR (('coronary' NEXT/2 ('disease*' OR 'syndrome')):ti) OR (('heart' NEXT/1 ('arrest*' OR 'attack*' OR 'disease*' OR 'failure*')):ti) | 1,182,686 |
| 2 | ‘cardiovascular disease’/de AND ([embase]/lim OR [embase classic]/lim OR [preprint]/lim) | 345,242 |
| 3 | ‘sudden cardiac death’/de AND ([embase]/lim OR [embase classic]/lim OR [preprint]/lim) | 25,544 |
| 4 | ‘heart disease’/de AND ([embase]/lim OR [embase classic]/lim OR [preprint]/lim) | 130,420 |
| 5 | ‘vascular disease’/de AND ([embase]/lim OR [embase classic]/lim OR [preprint]/lim) | 69,149 |
| 6 | ‘cerebrovascular disease’/de AND ([embase]/lim OR [embase classic]/lim OR [preprint]/lim) | 64,826 |
| 7 | #1 OR #2 OR #3 OR #4 OR #5 OR #6 | 1,648,863 |
| 8 | ‘Incidence’/de or ‘inciden*’:ab,ti AND ([embase]/lim OR [embase classic]/lim OR [preprint]/lim) | 1,643,580 |
| 9 | ‘Prevalence’/de or ‘prevalen*’:ab,ti AND ([embase]/lim OR [embase classic]/lim OR [preprint]/lim) | 1,512,678 |
| 10 | ‘Risk’/de or ‘Risk Factor’/de or ‘risk*’:ab,ti AND ([embase]/lim OR [embase classic]/lim OR [preprint]/lim) | 4,301,903 |
| 11 | 'disease management'/mj OR 'medication therapy management'/mj OR 'case management'/mj OR 'therapy'/mj OR 'management':ti OR 'treatment':ti OR 'therap*’:ti AND ([embase]/lim OR [embase classic]/lim OR [preprint]/lim) | 3,031,888 |
| 12 | (#8 OR #9 OR #10) NOT #11 | 5,507,669 |
| 13 | 'cohort analysis'/de OR (('cohort' NEXT/2 ('stud*' OR 'analy*')):ti,ab) OR 'longitudinal':ti,ab OR (('prospective' NEXT/2 ('stud*' OR 'analy*' OR 'data')):ti,ab) OR (('retrospective' NEXT/2 ('stud*' OR 'analy*' OR 'data')):ti,ab) AND ([embase]/lim OR [embase classic]/lim OR [preprint]/lim) | 2,507,752 |
| 14 | ‘Randomized controlled trial'/de or ‘Cross-sectional study’/de or ‘case control study’/de or ((‘cross-sectional’ NEXT/2 (‘study*’ OR ‘analy*’ OR ‘data’)):ti,ab) OR ‘randomi*ed control trial*’:ti,ab OR ((‘case’ NEXT/1 ‘control’ NEXT/2 (‘stud*’ or ‘analy*’ or ‘data’)):ti,ab) AND ([embase]/lim OR [embase classic]/lim OR [preprint]/lim) | 1,484,727 |
| 15 | ‘Systematic Review’/de OR ‘Meta Analysis’/de OR ‘Letter’/de OR ‘editorial’/de OR ‘clinical protocol’/de OR 'systematic review*':ti OR 'meta-analys*':ti OR ((‘meta’ NEXT/1 ‘analys*’):ti) OR ((‘letter’ OR ‘comment’ OR ‘editorial’ OR ‘protocol’):ti) AND ([embase]/lim OR [embase classic]/lim OR [preprint]/lim) | 2,503,454 |
| 16 | ‘Infant’/de OR ‘juvenile’/exp OR ((‘adolescen*’ or ‘child*’ or ‘schoolchild*’ or ‘infant*’ or ‘girl*’ or ‘boy*’ or ‘teen*’ or ‘youth*’ or ‘p?ediatr*’ or ‘puber*’):ti,ab) AND ([embase]/lim OR [embase classic]/lim OR [preprint]/lim) | 3,983,703 |
| 17 | #13 NOT (#14 OR #15 OR #16) | 1,739,752 |
| 18 | ‘Residence characteristics’/de or ‘Population density’/de or ‘City’/de or ‘Home environment’/de or ‘land use’/exp AND ([embase]/lim OR [embase classic]/lim OR [preprint]/lim) | 271,024 |
| 19 | ((‘Neighbo?rhood*’ or ‘communit*’ or ‘residence*’ or ‘built*’ or ‘contextual*’ or ‘geograph*’ or ‘urban’ or ‘rural’ or ‘environment*’ or ‘cit*’ or ‘home’) NEAR/2 (‘determinant*’ or ‘factor*’ or ‘environment*’ or ‘characteristic*’ or ‘exposure’ or ‘attribute*’ or ‘area’ or ‘region’ or ‘impact’ or ‘effect’ or ‘design’)):ti AND ([embase]/lim OR [embase classic]/lim OR [preprint]/lim) | 192,729 |
| 20 | ‘Health care facility’/de or ‘resource limited setting’/de AND ([embase]/lim OR [embase classic]/lim OR [preprint]/lim) | 65,564 |
| 21 | (((‘health service*’ or ‘health care’ or ‘care’ or ‘GP*’ or ‘general practice*’ or ‘hospital*’ or ‘secondary care’ or ‘primary care’ or ‘pharmacy’ or ‘pharmacist’ or ‘dentist’ or ‘leisure service*’ or ‘leisure cent*’ or ‘recreational cent*’) NEAR/2 (‘access’ or ‘access to’ or ‘quality’ or ‘quality of’ or ‘inaccess*’ or ‘lack*’ or ‘lack of’ or ‘environment*’ or ‘disparit*’)):ti,ab) AND ([embase]/lim OR [embase classic]/lim OR [preprint]/lim) | 174,925 |
| 22 | ‘social determinants of health’/de or ‘social aspects and related phenomena’/de or ‘Socioeconomic parameters’/de or ‘health disparity’/de or ‘poverty’/de or ‘social isolation’/de or ‘cultural deprivation’/de AND ([embase]/lim OR [embase classic]/lim OR [preprint]/lim) | 110,475 |
| 23 | (‘social determinant*’ or ‘socio?economic*’ or ‘disparit*’ or ‘poverty’ or ‘deprivation’ or ‘segregation*’ or ‘insecurity’ or ‘security’):ti AND ([embase]/lim OR [embase classic]/lim OR [preprint]/lim) | 91,816 |
| 24 | ‘Pollution’/de or ‘air pollution’/de or ‘exhaust gas’/de or ‘water pollution’/de or ‘light pollution’/de or ‘noise pollution’/de or ‘walkability’/de or ‘traffic and transport’/de AND ([embase]/lim OR [embase classic]/lim OR [preprint]/lim) | 167,355 |
| 25 | (‘pollut*’ or ‘air quality’):ti AND ([embase]/lim OR [embase classic]/lim OR [preprint]/lim) | 69,024 |
| 26 | (‘green space*’ or ‘blue space*’ or ‘walkability’):ti,ab OR (‘transport*’ or ‘transit’):ti AND ([embase]/lim OR [embase classic]/lim OR [preprint]/lim) | 166,732 |
| 27 | ‘food deprivation’/de or ‘food access’/de or ‘healthy food access’/de or ‘food availability’/de or ‘food desert’/de AND ([embase]/lim OR [embase classic]/lim OR [preprint]/lim) | 12,545 |
| 28 | ((‘fast?food*’ or ‘food*’ or ‘retail*’ or ‘grocery*’ or ‘supermarket*’ or ‘shop*’ or ‘nutrition*’ or ‘convenience*’ or ‘restaurant*’ or ‘nightlife*’ or ‘pub*’ or ‘bar*’ or ‘nightclub*’ or ‘off?license*’ OR ‘liquor store*’ or ‘tobacconist*’ or ‘gambling outlet*’) NEAR/2 (‘access’ or ‘access to’ or ‘quality’ or ‘quality of’ or ‘inaccess*’ or ‘lack*’ or ‘lack of’ or ‘environment*’ or ‘disparit*’)):ti,ab AND ([embase]/lim OR [embase classic]/lim OR [preprint]/lim) | 62,841 |
| 29 | #18 OR #19 OR #20 OR #21 OR #22 OR #23 OR #24 OR #25 OR #26 OR #27 OR #28 | 1,184,163 |
| 30 | ((‘national’ or ‘nationwide’ or ‘registry’ or ‘countrywide’ or ‘community’ or ‘population’ or ‘electronic health record*’):ti,ab,kw) or ‘electronic health record’/de AND ([embase]/lim OR [embase classic]/lim OR [preprint]/lim) | 3,590,042 |
| 31 | #7 AND #12 AND #17 AND #29 AND #30 | 2,577 |
| 32 | #31 AND [English]/lim | 2,548 |

**Table S5: Example of data extraction table**

| **Data extraction** |
| --- |
| Author |
| Year of publication |
| Location: continent |
| Location: country |
| Data source |
| Study period |
| Age (mean) |
| Sex (% male) |
| Outcome |
| Exposure domain |
| Exposure specific |
| Exposure measure |
| Exposure measurement method |
| Sample size |
| Event number |
| Result measure |
| Result value |
| Result lower confidence interval |
| Result upper confidence interval |
| Result unit increase measure |
| Result unit increase value |
| Adjustments for confounding |
| Notes |

**Table S6: Summary table of included studies**

| **Study ID** | **Region** | **Data source** | **Study period** | **Sample size** | **Outcome(s)** | **Exposure(s)** | **Exposure measurement method** | **Adjustments for confounding in the main model** | **Exclusion criteria for previous CVD** | **Notes** |
| --- | --- | --- | --- | --- | --- | --- | --- | --- | --- | --- |
| **Air Pollution** | | | | | | | | | | |
| Alexeeff (2018)[1] | United States | Kaiser Permanente Northern California healthcare data | 2010–2015 | 41,869 | CVD event*,  MI,  Stroke | NO2,  NO,  BC | 31 days of mobile data collection of roadways in study area over a 1 year period (05/2015–05/2016); 30-meter spatial resolution; extrapolated to daily and yearly average exposure. | age, ethnicity, sex, BMI, national deprivation index, smoking, diabetes, COPD, hypertension, hyperlipidemia, hypertensive medication, statin medication, neighbourhood socioeconomic status | Excluded those with previous MI, stroke of HF | Standardised HR unit increases |
| Atkinson (2013)[2] | England | The Clinical Practice Research Datalink | 2003–2007 | 836,557 | MI*,  Stroke,  Arrhythmia, HF | PM10,  NO2,  SO2,  O3 | Annual mean concentrations at 1x1km spatial resolution. Air dispersion modelling, (1) fixed level for 2002 and time-dependent for previous years exposure during study period. | age, sex, smoking, BMI, diabetes, hypertension, Index of Multiple Deprivation | Excluded those with previous MI, stroke, arrhythmia, HF (for each outcome) | Unit conversion (ug/m^3 to ppb) for NO2, SO2 and O3  Standardised HR unit increases |
| Avellaneda-Gomez (2022)[3] | Spain | CMBD database (health administration database) | 2016–2017 | 3,521,274 | Isch stroke* | PM2.5,  NO2,  BC,  Green space (NDVI) | Annual mean concentration. Modelled through land use regression models at 100m spatial resolution. Extrapolated using data from 2010 & 2015.  NDVI: satellite-based measure derived at 30x30m resolution. | age, gender, smoking, individual socioeconomic status, area-level socioeconomic status | Excluded those with previous cerebrovascular diseases | Unit conversion (ug/m^3 to ppb) for NO2  Standardised HR unit increases |
| Bai (2018)[4] | Canada | Ontario Population Health and Environment Cohort | 1996–2012 | 1,135,817 | HF*,  MI | Ultrafine particles,  NO2 | Derived using land use regression models using Mobile monitoring data for 2 weeks in 09/2010 and 1 week in 03/2011. 3-year moving averages estimated and linked to nearest postcode. | age, sex, immigrant status, employment, education, annual household income, hypertension, diabetes, previous MI or HF, COPD, asthma, cancer, UFP or NO2 | Excluded those with previous HF or MI | Standardised HR unit increases |
| Bai (2019)[5] | Canada | Ontario Population Health and Environment Cohort | 2001–2015 | 5,141,172 | HF*,  MI | PM2.5,  NO2,  O3 | Annual estimates using satellite retrievals of aerosol optical depth, global atmospheric chemistry transport model and geographically weighted regression models at a 1x1km spatial resolution. Modelled for years 1998–2012. | age, sex, geographic region, north/south indicator, neighbourhood deprivation, health care access, diabetes, hypertension, arrhythmia, CHF, COPD, asthma, cancer, smoking, alcohol drinking, obesity, and physical activity | Excluded those with previous HF or MI | Standardised HR unit increases |
| Chen (2022)[6] | Taiwan | Health and Welfare Data Science Center, Ministry of Health and Welfare database | 2010–2015 | 1,362,284 | Stroke*,  Isch stroke,  Haemor stroke | PM2.5,  SO2,  NO2,  NOx,  O3,  CO | Annual average of monitoring station within the residents administrative subdivision (city, township, district). Measures from 2010–2015 used. | age, sex, income level, urbanization level | Excluded those with previous stroke | Standardised HR unit increases |
| Gwon (2023)[7] | South Korea | National Health Insurance Service– National Sample Cohort database | 2008–2014 | 292,091 | PAD* | SO2,  NO2 | Hourly measurement from closest monitoring station to residence. Cumulative average used for air pollution exposure. Measured data from 2008–2014. | age, sex, economic status | Excluded those who were treated for: PAD, hypertension, diabetes, dyslipidaemia, angina, MI, stroke, and HF in the 5 years prior to baseline | Unit conversion (ppm to ppb) for SO2 and NO2  Standardised HR unit increases |
| Kim (2017)[8] | South Korea | National Health Insurance Service– National Sample Cohort database | 2007–2013 | 136,094 | CVD*,  MI,  HF,  Stroke,  Isch stroke,  Haemor stroke | PM2.5,  PM2.5–10,  CO,  SO2,  NO2,  O3 | Daily averages of nearest monitoring station to residence during study period. | age, sex, socioeconomic status, hypertension, diabetes mellitus, dyslipidemia, chronic renal failure, end-stage renal diseases, IHD, PAD, COPD, malignancy, BMI, fasting blood glucose, total cholesterol, hemoglobin | Excluded those with previous MI, HF, Stroke, Isch stroke, Haemor stroke | Standardised HR unit increases |
| Kim (2018)[9] | South Korea | National Health Insurance Service– National Sample Cohort database | 2009–2013 | 432,587 | AF* | PM2.5,  PM10,  SO2,  NO2,  CO,  O3 | Average annual concentration of nearest monitoring station to residence during study period. | age, sex, BMI, socioeconomic status, smoking status, temperature, humidity | Excluded those with previous AF  (Sensitivity analysis excluding those with further co-morbidities: HF, hypertension, stroke, MI) | Standardised HR unit increases |
| Noh (2019)[10] | South Korea | National Health Insurance Service– National Sample Cohort database | 2002–2013 | 62,676 | Haemor stroke* | PM2.5 | Annual mean of hourly monitored data in the district they lived from 2003–2013. | age, sex, insurance type, income decile, medical histories, BMI, smoking status, family medical history, moving residence | Excluded those with previous Haemor stroke | No conversion needed |
| Olaniyan (2022)[11] | Canada | The Canadian Institute of Health Information Discharge Abstract Database | 2006–2016 | 2,687,900 | MI*, Stroke | PM2.5,  NO2,  O3 | Annual estimates using satellite retrievals of aerosol optical depth, global atmospheric chemistry transport model and geographically weighted regression models at a 1x1km spatial resolution. Data from 1998–2012 used for estimating. | age, sex, immigration status, Socioeconomic status, occupational class, marital status, education, income deciles, indigenous status, visible minority status, employment status, Canadian marginalization indices, urbanisation, community size | Excluded those with previous MI or stroke (up to 13 years before baseline) | Standardised HR unit increases |
| Poulsen (2023)[12] | Denmark | Danish National Patient Register | 2005–2017 | 1,964,702 | MI* | PM2.5,  UFP,  EC,  NO2 | Modelled monthly mean concentrations, calculated into running mean exposure at 1,5, and 10y. Modelled at 1x1km spatial resolution. Modelled exposure for years 1995–2017. | age, sex, calendar year, marital status, education, occupational status, ethnicity, personal and household income, area level % of population: living in social housing, being sole providers, of non-Western origin, having low income, being unemployed, having blue-collar work, having only basic education, and having a criminal record | Excluded those with previous MI | Unit conversion (ug/m^3 to ppb) for NO2  Standardised HR unit increases |
| Shin (2019)[13] | Canada | Ontario Population Health and Environment Cohort | 2001–2015 | 5,071,956 | AF*,  Stroke | PM2.5,  NO2,  O3,  Ox | Satellite observations of aerosol optical depth; land use regression models; annual averages at 1x1km spatial resolution, 1998–2012 | age, sex, income quintile, Area level indicators: education, unemployment rate, proportion of recent immigrants, north/south, urban/rural | Excluded those with previous AF or stroke | Standardised HR unit increases |
| Vanoli (2024)[14] | United Kingdom | UK Biobank | 2006–2021 | 377,736 | Composite CVD*, MI, Stroke, HF, AF | PM2.5 | Hybrid spatio-temporal machine learning model predicted daily levels at 1x1km spatial resolution. Daily levels modelled for period 2003–2021. | assessment centre, sex, year of birth, ethnic background, education level, household income, employment status, smoking status, packs of cigarettes per year, average alcohol intake per week, waist-to-hip ratio, physical activity, living alone. Area-level covariates: Townsend Deprivation Index measured in 2010, urban–rural classification, greenness percentage around residential address | Prevalent CVD cases at baseline | Standardised HR unit increases |
| Wang (2021)[15] | United Kingdom | UK Biobank | (2006–2010) – 2018 | 432,530 | HF* | PM2.5,  PM10,  PM2.5–10,  NO2,  NOx | Annual average concentration estimated using land-use regression models. Data taken from years 2005, 2006, 2007 and 2010. | age, sex, ethnicity, UK Biobank assessment centre, Townsend Deprivation index, alcohol consumption, smoking status, BMI, physical activity, healthy diet score, diabetes, hypertension, blood pressure | Excluded those with previous HF, AF or CHD | Unit conversion (ug/m^3 to ppb) for NO2  Standardised HR unit increases |
| Zhang (2019)[16] | Taiwan | The National Health Institute Research Database | 2000–2013 | 283,666 | Isch stroke* | Hydrocarbons,  Non-methane hydrocarbons | Patients residential postcode linked to nearest air pollutant monitoring station. Daily average concentration used. Data collected from 07/1993–12/2013. | age, gender, insurance amount, Charlson Comorbidity Index, hypertension, diabetes, hyperlipidaemia, CHD, PAD, season, and ambient temperature | Excluded those with previous Isch stroke | No conversion needed |
| Zhang (2024)[17] | United Kingdom | UK Biobank | 2006–2020 | 285,009 | IHD*,  Stroke,  AF,  HF | PM2.5,  PM10,  NO2,  NOx | Annual mean concentration of air pollutants at 1x1km spatial resolution linked to residential postcode during study period. | age and sex, relative humidity, ambient temperature, ethnicity, physical activity, income and education. | Excluded those with history of CVD and hypertension | Unit conversion (ug/m^3 to ppb) for NO2  Standardised HR unit increases |
| **Noise Pollution** | | | | | | | | | | |
| Bai (2020)[18] | Canada | Ontario Population Health and Environment Cohort | 2001–2015 | 1,005,214 | MI*,  HF | Average 24-hour road traffic noise exposure (dBA)  Average night-time exposure (dBA) | Noise propagation model estimating noise from road traffic only, on a 10x10m resolution. Extracted estimates of an A-weighted equivalent sound pression level over a 24-h period. 3-year moving average of estimates calculated i.e. exposure for 2001 was estimated as the mean exposure for the years 1998–2000. | age, sex, neighbourhood level: recent immigrants, unemployment rate, education, annual household income, diabetes, hypertension, stroke, asthma, COPD, cancer, smoking, BMI | Excluded those with previous MI or HF | Standardised HR unit increases  Converted HR to RR |
| Magnoni (2021)[19] | Italy | Agency for Health Protection of Milan (data warehouse systems) | 2011–2018 | 1,087,110 | CVD event,  MI,  Isch stroke,  Haemor stroke | Road traffic noise (dBA) | Noise predictive model (integrates acoustic characterisation of road infrastructure, territory morphology, land use, and buildings. Mean annual estimates of A-weighted equivalent noise level over a whole day for the year 2012. | age, sex, citizenship and Italian Deprivation Index (IDI) | Excluded those with previous MI, Isch stroke, Haemor stroke | Unable to include in meta-analysis due to categorised outcome measure |
| Poulsen (2019)[20] | Denmark | Danish National Patient Register | 1982–2013 | 717,453 | MI,  Stroke | Wind turbine noise | Noise propagation modelling for all eligible buildings taking into account hourly estimates of wind speed and direction between 1982–2013. A-weighted noise exposure level calculated for each dwelling. | age, sex, calendar year, personal income, education, marital status, employment, area-level socioeconomic status, type of dwelling, traffic load in 500-m radius and distance to major road | Excluded those with previous MI, stroke | Not included in meta-analysis due to limited no. of studies with this exposure measure |
| Poulsen (2024)[21] | Denmark | Danish National Patient Register | 2005–2017 | 1,964,702 | MI* | Road traffic noise,  PM2.5,  UFP,  EC,  NO2,  Available green space | Noise predictive modelling for most and least exposed façade for all Danish dwellings (input from road links, road types, average daily traffic load, travel speed and light/heavy vehicle distribution). Modelled for the years 2005, 2010 and 2015 then linearly interpolated for all other years (2005–2017). Annual mean estimates. | educational levels, occupational status, marital status, country of origin, personal income, and household income. proportion of inhabitants (within parishes) with only basic education, with manual labour, with income in the lowest quartile, living in social housing, living in single-parent households, with a criminal record, and with non-Western background. | Excluded those with previous MI | Standardised HR unit increases  Converted HR to RR |
| Seidler (2016)[22] | Germany | Three state health insurers administrative data (Darmstadt district) | 2006–2010 | 854,366 | MI* | Average 24 hour road, rail and aircraft noise exposure | 24 hour continuous average noise level, for the reference year 2005. Address specific noise modelling. | age, sex, education, occupation, rate of over 65 and receiving state benefits. | None stated | Converted OR to RR |
| Sørensen (2021)[23] | Denmark | Danish National Patient Register | 2000–2017 | 3,620,000 | Stroke* | Average 24 hour road and rail traffic noise | Road traffic noise modelled (Nordic prediction method) for the years 1995, 2000, 2005, 2010 and 2015 for all Danish residential addresses. Equivalent a-weighted sound-pressure level for 24-hours. | age, sex, calendar year, civil status, income, region of origin, occupational status, railway noise, area level: low income, basic education, unemployment, manual labour, single-parent, criminal records | None stated | No conversion needed |
| Thacher (2022a)[24] | Denmark | Danish National Patient Register | 2000–2017 | 3,604,968 | AF* | 1, 5 and 10 year average road, rail and aircraft traffic noise | Road traffic noise modelled (Nordic prediction method) for the years 1995, 2000, 2005, 2010 and 2015 for all Danish residential addresses. Equivalent a-weighted sound-pressure level for 24-hours. | age, sex, calendar year, civil status, income, country of origin, occupational status, green space, area level: low income, basic education, unemployment, manual labour, railway and aircraft noise | Excluded those with previous AF | No conversion needed |
| Thacher (2022b)[25] | Denmark | Danish National Patient Register | 2005–2017 | 2,538,395 | CHD*,  MI,  Angina,  HF | 1, 5 and 10 year average road, rail and aircraft traffic noise | Road traffic noise modelled (Nordic prediction method) for the years 1995, 2000, 2005, 2010 and 2015 for all Danish residential addresses. Equivalent a-weighted sound-pressure level for 24-hours. | age, sex, calendar year, civil status, income, country of origin, occupational status, educational level, green space, area level: low income, basic education, unemployment, sole-provider families, manual labour, railway and aircraft noise | Excluded those with previous CHD, MI, Angina, HF | Converted HR to RR |
| Thacher (2024)[26] | Denmark, Sweden, Finland | 11 pooled prospective Nordic cohorts | Up to 2017 | 161,115 | AF* | 5-year exposure to road, rail, aircraft noise | Nordic prediction method to measure road traffic noise for each residential address. A-weighted sound pressure levels. Measured as time weighted mean for previous 5 years. | adjusted for age, cohort (strata), sex and calendar year, educational level, marital status, area–income, and other noise sources (road, railway and aircraft noise), smoking status and physical activity, time–weighted PM2.5 exposure (1– or 5-year) | CVD free at baseline | Converted HR to RR |
| **Green and blue space** | | | | | | | | | | |
| Seo (2019)[27] | South Korea | National Health Insurance Service– National Sample Cohort database | 2006–2013 | 351,409 | CVD | Green space (% cover) | Ministry of Land, Infrastructure and Transport provided measures (area and number) of land lots which included parks and green space. Used data collected in 2005. Exposure measured as the proportion (%) of urban green space land lots for each district. | age, sex, income, disability, and Charlson comorbidity index | Excluded those with previous CVD | Not included in meta-analysis due to limited no. of studies with this exposure measure |
| Wang (2019)[28] | United States | US Centers for Medicare & Medicaid Services Master Beneficiary Summary File for Miami-Dade County | 2010–2011 | 249,405 | MI,  Isch stroke,  CHD,  HF,  AF | Green space (NDVI) | US National Aeronautics and Space Administration Advanced Spaceborne Thermal Emission and Reflection Radiometer satellite imagery at 15x15m spatial resolution used to calculate NDVI. | age, sex, ethnicity, neighbourhood median household income, diabetes, hypertension, hyperlipidemia | None stated | Not included in meta-analysis due to limited no. of studies with this exposure measure |
| Xiao-Dong (2022)[29] | United Kingdom | UK Biobank | (2007–2010)–2017 | 377,340 | MI | Blue space (distance to coastline) | Residential distance to the coastline (km). Euclidean distance raster from the coastline calculated for small grid size, values from grid allocated to participant home location grid references. | age, gender, ethnicity, education, income, employment, physical activity, mental health status, health rating, smoking, drinking, BMI, waist to hip ratio, grip strength, and area-based socioeconomic status, family history of heart diseases, the medication use of aspirin, antihypertension medicine and lip-lowing medicine, previous diagnosis of hypertension and diabetes, air pollution, noise pollution, dietary, sleep pattern, and area population density. | Excluded those with previous CHD, stroke | Not included in meta-analysis due to limited no. of studies with this exposure measure |
| **Socioeconomic** | | | | | | | | | | |
| Essien (2022)[30] | United States | Northwestern Medicine Enterprise Data Warehouse | 2005–2018 | 28,858 | AF* | Neighbourhood-level poverty | US census tract geographic area used as proxy for ‘neighbourhood’. Neighbourhood poverty quantified as the proportion of people in a given census tract living below the US-defined poverty threshold. | Age, sex, ethnicity, BMI, blood pressure, glucose level, hypertension, diabetes, smoking, neighbourhood poverty | Excluded those with previous AF, coronary artery disease, stroke/ cerebrovascular disease, PAD, HF, presence of a pacemaker | Converted OR to RR |
| Gwon (2020)[31] | South Korea | National Health Insurance Service– National Sample Cohort database | 2009–2013 | 356,125 | IHD* | Community-level socioeconomic inequality | Community level SES defined as the local income for the residential area in which participants lived in 2009. | age, sex, smoking, BMI, individual economic status, diabetes, hypertension, dyslipidemia, PAD and stroke | Excluded those with previous IHD | Converted HR to RR |
| Lönn (2019)[32] | Sweden | Swedish medical registers | (2003–2007)–(2008–2012) | 3,140,657 | CHD | Accumulated neighbourhood deprivation | Based on Small Areas for Market Statistics (avg 1000 inhabitants) obtained from Statistics Sweden. Aggregated measure based on proportion of people in area with low income, low education, unemployment and receipt of social welfare. | education, place of residence, cohabiting status, hypertension, HF, valvular heart disease, diabetes, IHD, PAD, hyperthyroidism, COPD, chronic kidney disease, alcoholism, obesity and CHD. | Excluded those with previous CHD | Unable to include in meta-analysis due to categorised outcome measure |
| Pujades-Rodriquez (2014)[33] | United Kingdom | Clinical Practice Research Datalink | 1997–2010 | 1,937,360 | CVD | Index of multiple deprivation | Used index of multiple deprivation (common composite indicator in the UK calculated from 38 indicators across 7 domains: income, employment, health and disability, education, skills and training, barriers to housing services, crime and living environment) calculated at lower layer super output area (avg 1500 inhabitants) linked to residential postcode. Measured in 2007. | sex, age, ethnicity, diabetes, smoking, BMI, systolic blood pressure, total and high-density lipoprotein cholesterol, and medication use (blood pressure lowering drugs, statins, oestrogen oral contraceptives and hormone replacement therapy). | Excluded those with previous CVD | Unable to include in meta-analysis due to categorised outcome measure |
| Rethy (2021)[34] | United States | Northwestern Memorial Enterprise Data Warehouse | 2005–2013 | 28,858 | HF* | Neighbourhood-level poverty | Proportion of people living below the US defined poverty threshold using data from 2007–2011 using census tract geographic areas. | age, sex, ethnicity, hypertension, deprivation, BMI, smoking | Excluded those with previous IHD, HF, PAD, stroke, presence of a pacemaker | Converted OR to RR |
| Winkleby (2007)[35] | Sweden | Swedish Hospital Discharge Register | 1996–2000 | 3,755,108 | CHD* | Neighbourhood-level deprivation | Based on Small Areas for Market Statistics (avg 1000 inhabitants) obtained from Statistics Sweden. Aggregated measure based on proportion of people in area with low income, low education, unemployment and receipt of social welfare. Measures taken in 1995. | age, marital status, family income, education, immigration status, mobility, and urban/rural status | Excluded those with previous CHD (up to 10 years prior) | Converted OR to RR |
| **Food and healthcare environment** | | | | | | | | | | |
| Hamano (2013)[36] | Sweden | Swedish Hospital Discharge Register | 2005–2007 | 4,309,674 | Stroke | Fast-food restaurant, bars/pubs, health care facilities, and physical activity facilities availability | Based on Small Areas for Market Statistics (avg 1000 inhabitants). Counts for ‘health damaging’ establishment per small area for market statistics geographic area. Measures taken in November 2005. | neighbourhood level deprivation, individual income, age | Excluded those with previous stroke | Not included in meta-analysis: unable to synchronise exposure measure across other studies. |
| Kawakami (2011)[37] | Sweden | Swedish Hospital Discharge Register | 2005–2007 | 2,165,000 | CHD | Fast-food restaurant, bars/pubs, health care facilities, and physical activity facilities availability | Based on Small Areas for Market Statistics (avg 1000 inhabitants). Counts for ‘health damaging’ establishment per small area for market statistics geographic area. Measures taken in November 2005. | neighbourhood level deprivation, individual income, age | Excluded those with previous CHD (up to 5 years prior) | Not included in meta-analysis: unable to synchronise exposure measure across other studies. |
| Pinho (2024)[38] | The Netherlands | Dutch hospital data & registry data from Statistics Netherlands | 2004–2018 | 4,641,435 | CVD,  CHD,  Stroke,  HF | Food environment healthiness index (FEHI),  Density of:  Local food shops,  Fast-food outlets,  Food delivery outlets,  Restaurants,  Supermarkets,  Convenience stores | Type and location of retailers obtained from commercial database. Point location of each food retailer bi-annually from 2004 to 2018 used to calculate the time-varying densities of outlets. Kernal density method used to calculate grid-based kernal density distances in a circle with a 1000m radius using 25x25m grid cell sizes. These calculated distances assigned to the corresponding residential location. | age, sex, migration background, household composition, income, marital status, and neighbourhood urbanization level | Excluded those with ant CVD outcome at baseline. | Not included in meta-analysis: unable to synchronise exposure measure across other studies. |
| Poelman (2020)[39] | The Netherlands | Several Dutch national registers (inc. hospital discharge register) | 2009–2010 | 2,472,004 | CVD,  CHD,  Stroke,  HF | No. of Fast-food restaurants (0, 1, 2, >=3) within 500m/ 1000m/ 3000m | Fast food density defined as the number of fast-food restaurants within network buffers around an address. Calculated using ArcGIS. | age, sex, ethnicity, marital status, Charlson comorbidity index, neighbourhood-level income, population density | None stated | Not included in meta-analysis: unable to synchronise exposure measure across other studies. |

* Primary outcome used for composite CVD measure in the meta-analysis.

**References**

1. Alexeeff, S.E., et al., *High-resolution mapping of traffic related air pollution with Google street view cars and incidence of cardiovascular events within neighborhoods in Oakland, CA.* Environmental Health: A Global Access Science Source, 2018. **17**(1).

2. Atkinson, R.W., et al., *Long-term exposure to outdoor air pollution and incidence of cardiovascular diseases.* Epidemiology, 2013. **24**(1): p. 44-53.

3. Avellaneda-Gómez, C., et al., *Air pollution and surrounding greenness in relation to ischemic stroke: A population-based cohort study.* Environment International, 2022. **161**.

4. Bai, L., et al., *Associations of Long-Term Exposure to Ultrafine Particles and Nitrogen Dioxide With Increased Incidence of Congestive Heart Failure and Acute Myocardial Infarction.* American Journal of Epidemiology, 2019. **188**(1): p. 151-159.

5. Bai, L., et al., *Exposure to ambient air pollution and the incidence of congestive heart failure and acute myocardial infarction: A population-based study of 5.1 million Canadian adults living in Ontario.* Environment International, 2019. **132**: p. 105004.

6. Chen, P.C., et al., *A cohort study evaluating the risk of stroke associated with long-term exposure to ambient fine particulate matter in Taiwan.* Environ Health, 2022. **21**(1): p. 43.

7. Gwon, J.G., et al., *Exposure to Long-Term Air Pollution and Incidence of Peripheral Arterial Disease in the General Population: A Korean National Population-Based Retrospective Cohort Study.* Angiology, 2023. **74**(8): p. 721-727.

8. Kim, H., et al., *Cardiovascular Effects of Long‐Term Exposure to Air Pollution: A Population‐Based Study With 900 845 Person‐Years of Follow‐up.* Journal of the American Heart Association, 2017. **6**(11): p. e007170.

9. Kim, I.S., et al., *Particulate matter air pollution and incident atrial fibrillation in a general healthy population: A nationwide cohort study.* Europace, 2018. **20**: p. i15.

10. Noh, J., et al., *Long-term Effects of Cumulative Average PM2.5 Exposure on the Risk of Hemorrhagic Stroke.* Epidemiology, 2019. **30**: p. S90-S98.

11. Olaniyan, T., et al., *Ambient air pollution and the risk of acute myocardial infarction and stroke: A national cohort study.* Environmental Research, 2022. **204**.

12. Poulsen, A.H., et al., *Source-Specific Air Pollution Including Ultrafine Particles and Risk of Myocardial Infarction: A Nationwide Cohort Study from Denmark.* Environmental Health Perspectives, 2023. **131**(5).

13. Shin, S., et al., *Ambient Air Pollution and the Risk of Atrial Fibrillation and Stroke: A Population-Based Cohort Study.* Environmental Health Perspectives, 2019. **127**(8): p. 87009.

14. Vanoli, J., et al., *Association between long-term exposure to low ambient PM2.5 and cardiovascular hospital admissions: A UK Biobank study.* Environment International, 2024. **192**.

15. Wang, M., et al., *Joint exposure to various ambient air pollutants and incident heart failure: a prospective analysis in UK Biobank.* Eur Heart J, 2021. **42**(16): p. 1582-1591.

16. Zhang, H.W., et al., *Long-term ambient hydrocarbons exposure and incidence of ischemic stroke.* PLoS ONE, 2019. **14**(12).

17. Zhang, S., et al., *Associations between air pollution and the risk of first admission and multiple readmissions for cardiovascular diseases.* Heart, 2024. **110**(5): p. 337-345.

18. Bai, L., et al., *Exposure to road traffic noise and incidence of acute myocardial infarction and congestive heart failure: A population-based cohort study in Toronto, Canada.* Environmental Health Perspectives, 2020. **128**(8): p. 1-9.

19. Magnoni, P., R. Murtas, and A.G. Russo, *Residential exposure to traffic-borne pollution as a risk factor for acute cardiocerebrovascular events: a population-based retrospective cohort study in a highly urbanized area.* International Journal of Epidemiology, 2021. **50**(4): p. 1160-1171.

20. Poulsen, A.H., et al., *Long-term exposure to wind turbine noise and risk for myocardial infarction and stroke: A nationwide cohort study.* Environmental Health Perspectives, 2019. **127**(3).

21. Poulsen, A.H., et al., *Concomitant exposure to air pollution, green space and noise, and risk of myocardial infarction: a cohort study from Denmark.* European Journal of Preventive Cardiology, 2024. **31**(1): p. 131-141.

22. Seidler, A., et al., *Myocardial Infarction Risk Due to Aircraft, Road, and Rail Traffic Noise.* Dtsch Arztebl Int, 2016. **113**(24): p. 407-14.

23. Sørensen, M., et al., *Transportation noise and risk of stroke: A nationwide prospective cohort study covering Denmark.* International Journal of Epidemiology, 2021. **50**(4): p. 1147-1156.

24. Thacher, J.D., et al., *Long-term exposure to transportation noise and risk for atrial fibrillation: A Danish nationwide cohort study.* Environmental Research, 2022. **207**: p. 112167.

25. Thacher, J.D., et al., *Exposure to transportation noise and risk for cardiovascular disease in a nationwide cohort study from Denmark.* Environmental Research, 2022. **211**: p. 113106.

26. Thacher, J.D., et al., *Residential exposure to transportation noise and risk of incident atrial fibrillation: a pooled study of 11 prospective Nordic cohorts.* The Lancet Regional Health - Europe, 2024. **46**.

27. Seo, S., et al., *Association between urban green space and the risk of cardiovascular disease: A longitudinal study in seven Korean metropolitan areas.* Environment International, 2019. **125**: p. 51-57.

28. Wang, K., et al., *Relationship of Neighborhood Greenness to Heart Disease in 249 405 US Medicare Beneficiaries.* Journal of the American Heart Association, 2019. **8**(6): p. e010258.

29. Xiao-Dong, Z., et al., *Association of Residential Proximity to the Coast With Incident Myocardial Infarction: A Prospective Cohort Study.* Front Cardiovasc Med, 2022. **9**: p. 752964.

30. Essien, U.R., et al., *Association Between Neighborhood-Level Poverty and Incident Atrial Fibrillation: a Retrospective Cohort Study.* Journal of General Internal Medicine, 2022. **37**(6): p. 1436-1443.

31. Gwon, J.G., J. Choi, and Y.J. Han, *Community-level socioeconomic inequality in the incidence of ischemic heart disease: a nationwide cohort study.* BMC Cardiovascular Disorders, 2020. **20**(1): p. 87.

32. Lönn, S.L., et al., *Accumulated neighbourhood deprivation and coronary heart disease: A nationwide cohort study from Sweden.* BMJ Open, 2019. **9**(9).

33. Pujades-Rodriguez, M., et al., *Socioeconomic deprivation and the incidence of 12 cardiovascular diseases in 1.9 million women and men: Implications for risk prediction and prevention.* PLoS ONE, 2014. **9**(8).

34. Rethy, L.B., et al., *Neighborhood Poverty and Incident Heart Failure: an Analysis of Electronic Health Records from 2005 to 2018.* Journal of General Internal Medicine, 2021. **36**(12): p. 3719-3727.

35. Winkleby, M., K. Sundquist, and C. Cubbin, *Inequities in CHD incidence and case fatality by neighborhood deprivation.* Am J Prev Med, 2007. **32**(2): p. 97-106.

36. Hamano, T., et al., *Neighbourhood Environment and Stroke: A Follow-Up Study in Sweden.* PLOS ONE, 2013. **8**(2): p. e56680.

37. Naomi, K., L. Xinjun, and S. Kristina, *Health-promoting and health-damaging neighbourhood resources and coronary heart disease: a follow-up study of 2 165 000 people.* Journal of Epidemiology and Community Health, 2011. **65**(10): p. 866.

38. Pinho, M.G.M., et al., *Time-Varying Exposure to Food Retailers and Cardiovascular Disease Incidence and Mortality in the Netherlands: A Nationwide Prospective Cohort Study.* Circulation, 2024. **149**.

39. Poelman, M., et al., *Relations between the residential fast-food environment and the individual risk of cardiovascular diseases in The Netherlands: A nationwide follow-up study.* European Journal of Preventive Cardiology, 2020. **25**(13): p. 1397-1405.

**Table S7: Results of assessment for risk of bias (ROBINS-E)**

| **Study ID** | **Bias due to confounding** | **Bias due to measurement of exposure** | **Bias due to selection of participants** | **Bias due to post-exposure interventions** | **Bias due to missing data** | **Bias due to measurement of the outcome** | **Bias due to selection of the reported results** | **Overall risk of bias judgement** |
| --- | --- | --- | --- | --- | --- | --- | --- | --- |
| Alexeeff (2018) | Some concerns | Low risk | Low risk | Low risk | Some concerns | Low risk | Low risk | Some concerns |
| Atkinson (2013) | Some concerns | Some concerns | Low risk | Low risk | Low risk | Some concerns | Low risk | Some concerns |
| Avellaneda-Gómez (2022) | Low risk | Some concerns | Some concerns | Low risk | Some concerns | Some concerns | Low risk | Some concerns |
| Bai (2018) | Some concerns | Some concerns | Low risk | Low risk | Low risk | Low risk | Low risk | Some concerns |
| Bai (2019) | Some concerns | Some concerns | Low risk | Low risk | Low risk | Low risk | Low risk | Some concerns |
| Bai (2020) | Some concerns | Some concerns | Low risk | Low risk | Some concerns | Low risk | Low risk | Some concerns |
| Chen (2022) | Some concerns | Some concerns | Low risk | Low risk | Some concerns | Low risk | Low risk | Some concerns |
| Essien (2022) | Some concerns | Some concerns | Some concerns | Low risk | Some concerns | Low risk | Low risk | Some concerns |
| Gwon (2020) | Low risk | Some concerns | Some concerns | Low risk | High risk | Low risk | Low risk | Some concerns |
| Gwon (2023) | Some concerns | Some concerns | Low risk | Low risk | High risk | Low risk | Low risk | Some concerns |
| Hamano (2013) | High risk | Some concerns | Low risk | Low risk | Some concerns | Some concerns | Low risk | High risk |
| Kim (2017) | Some concerns | Some concerns | Low risk | Low risk | Some concerns | Some concerns | Low risk | Some concerns |
| Kim (2018) | Some concerns | Some concerns | Some concerns | Low risk | Some concerns | Some concerns | Low risk | Some concerns |
| Lönn (2019) | Some concerns | Some concerns | Some concerns | Low risk | Some concerns | Some concerns | Low risk | Some concerns |
| Magnoni (2021) | Some concerns | Some concerns | Low risk | Low risk | Some concerns | Some concerns | Low risk | Some concerns |
| Kawakami (2011) | High risk | Some concerns | Low risk | Low risk | Low risk | Some concerns | Low risk | Some concerns |
| Noh (2019) | Some concerns | Some concerns | Low risk | Low risk | Some concerns | Some concerns | Low risk | Some concerns |
| Olaniyan (2022) | Some concerns | Some concerns | Some concerns | Low risk | Some concerns | Some concerns | Low risk | Some concerns |
| Pinho (2024) | Some concerns | Low risk | Low risk | Low risk | Some concerns | Some concerns | Low risk | Some concerns |
| Poelman (2020)* | Some concerns | Some concerns | Some concerns | Low risk | Some concerns | Some concerns | Low risk | Some concerns |
| Poulsen (2019) | Some concerns | Some concerns | Some concerns | Low risk | Some concerns | Some concerns | Low risk | Some concerns |
| Poulsen (2023) | Some concerns | Some concerns | Low risk | Low risk | Low risk | Some concerns | Low risk | Some concerns |
| Poulsen (2024) | Some concerns | Some concerns | Low risk | Low risk | Low risk | Some concerns | Low risk | Some concerns |
| Pujades-Rodriquez (2014) | Some concerns | Some concerns | Some concerns | Low risk | Some concerns | Some concerns | Low risk | Some concerns |
| Rethy (2021) | Some concerns | Some concerns | Some concerns | Low risk | Low risk | Some concerns | Low risk | Some concerns |
| Seidler (2016)* | High risk | Some concerns | Some concerns | Low risk | High risk | Some concerns | Low risk | High risk |
| Seo (2019) | Some concerns | Some concerns | Some concerns | Low risk | Low risk | Some concerns | Low risk | Some concerns |
| Shin (2019) | Some concerns | Some concerns | Low risk | Low risk | Some concerns | Some concerns | Low risk | Some concerns |
| Sørensen (2021)* | High risk | Some concerns | Low risk | Low risk | Some concerns | Some concerns | Low risk | High risk |
| Thacher (2022a) | Some concerns | Some concerns | Low risk | Low risk | Some concerns | Some concerns | Low risk | Some concerns |
| Thacher (2022b) | Some concerns | Some concerns | Low risk | Low risk | Some concerns | Some concerns | Low risk | Some concerns |
| Thacher (2024) | Some concerns | Some concerns | High risk | Low risk | Some concerns | Some concerns | Low risk | Some concerns |
| Vanoli (2024) | Some concerns | Some concerns | Some concerns | Low risk | Low risk | Some concerns | Low risk | Some concerns |
| Wang (2019) | High risk | Some concerns | Low risk | Low risk | Some concerns | Some concerns | Low risk | High risk |
| Wang (2021)* | Some concerns | Some concerns | Low risk | Low risk | Some concerns | Some concerns | Low risk | Some concerns |
| Winkleby (2007) | High risk | Low risk | Some concerns | Low risk | Some concerns | Low risk | Low risk | Some concerns |
| Xiao-Dong (2022) | Some concerns | Low risk | Low risk | Low risk | Low risk | Low risk | Low risk | Low risk |
| Zhang (2019) | Some concerns | Some concerns | Low risk | Low risk | Some concerns | Some concerns | Low risk | Some concerns |
| Zhang (2024) | Some concerns | Some concerns | Some concerns | Low risk | Low risk | Some concerns | Low risk | Some concerns |

*did not state any exclusion criteria based on previous CVD (ensuring incident cases).

**Figure S1: A funnel plot for air pollutant NO2 demonstrating publication bias**


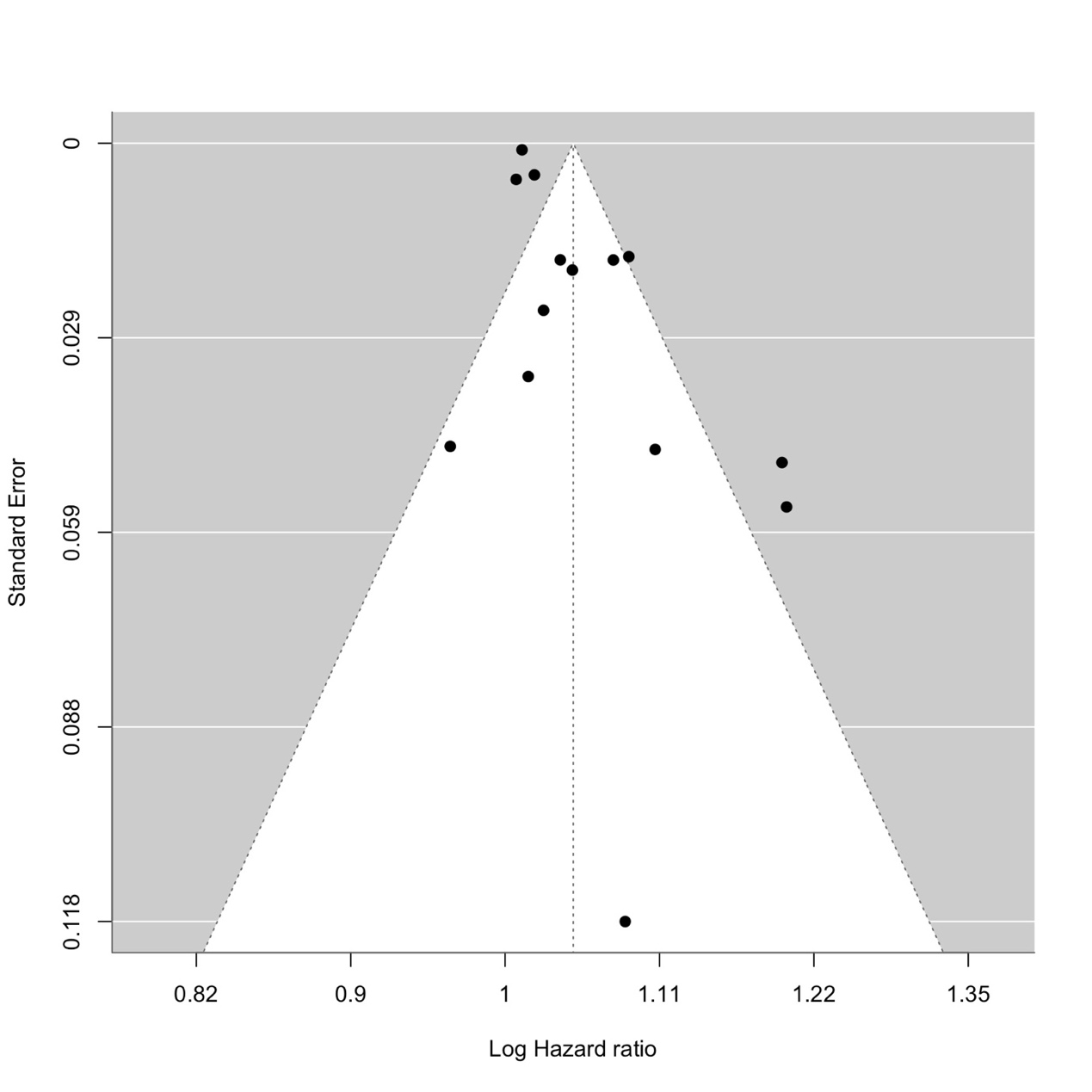


**Figure S2: Forest plot NO2 meta-analysis**

**
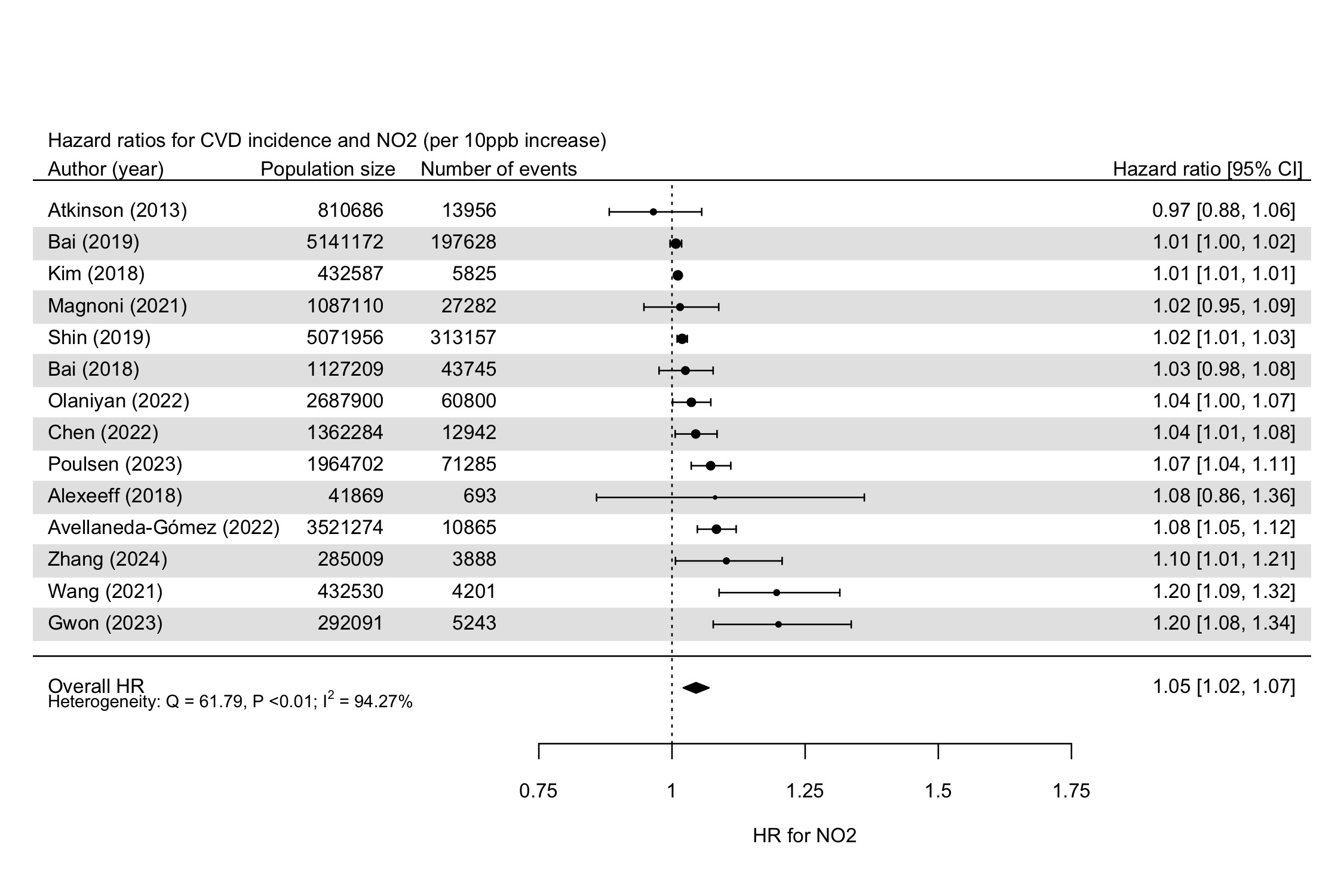
**

**Figure S3: Forest plot PM2.5 meta-analysis**

**
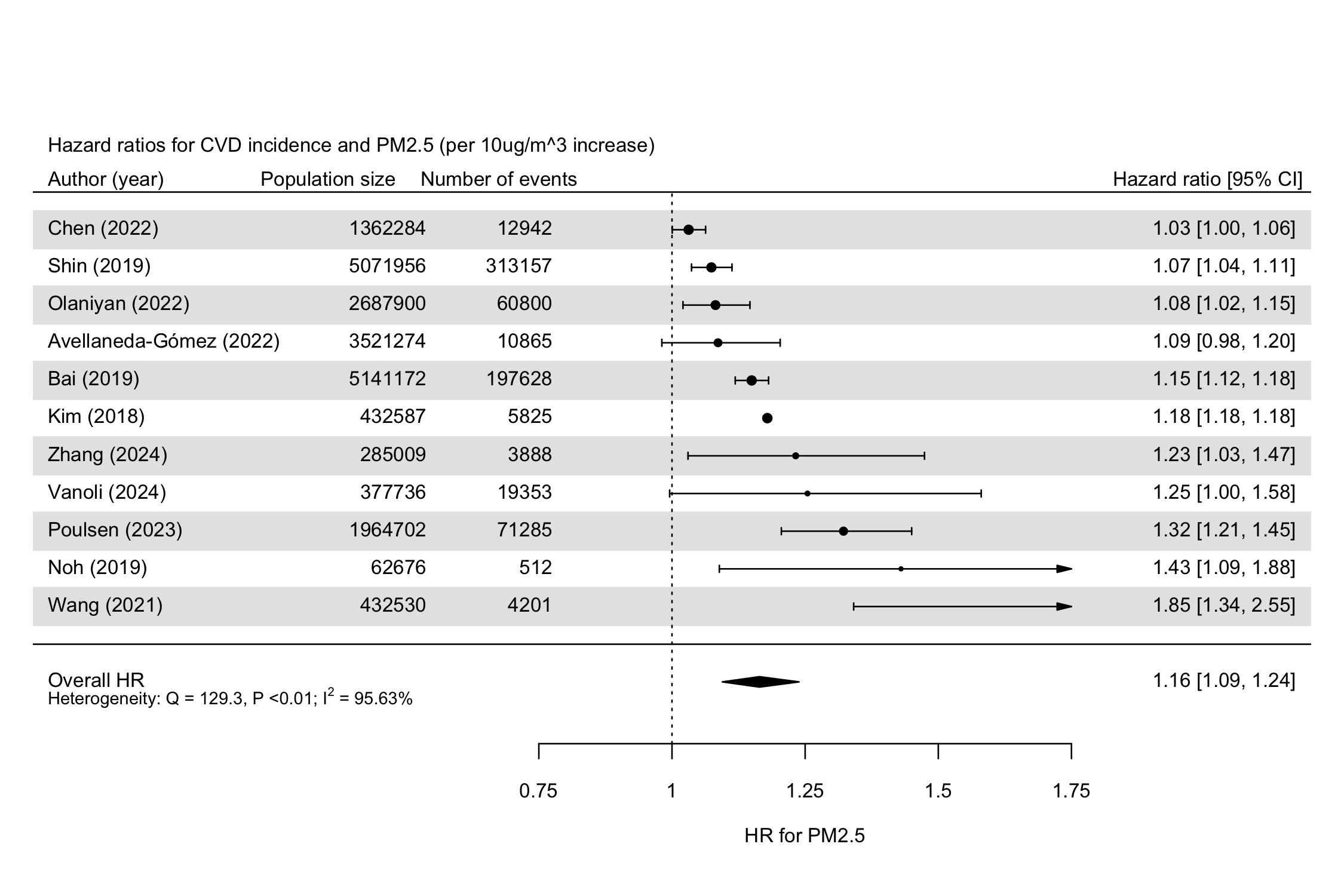
**

**Figure S4: Forest plot PM10 meta-analysis**

**
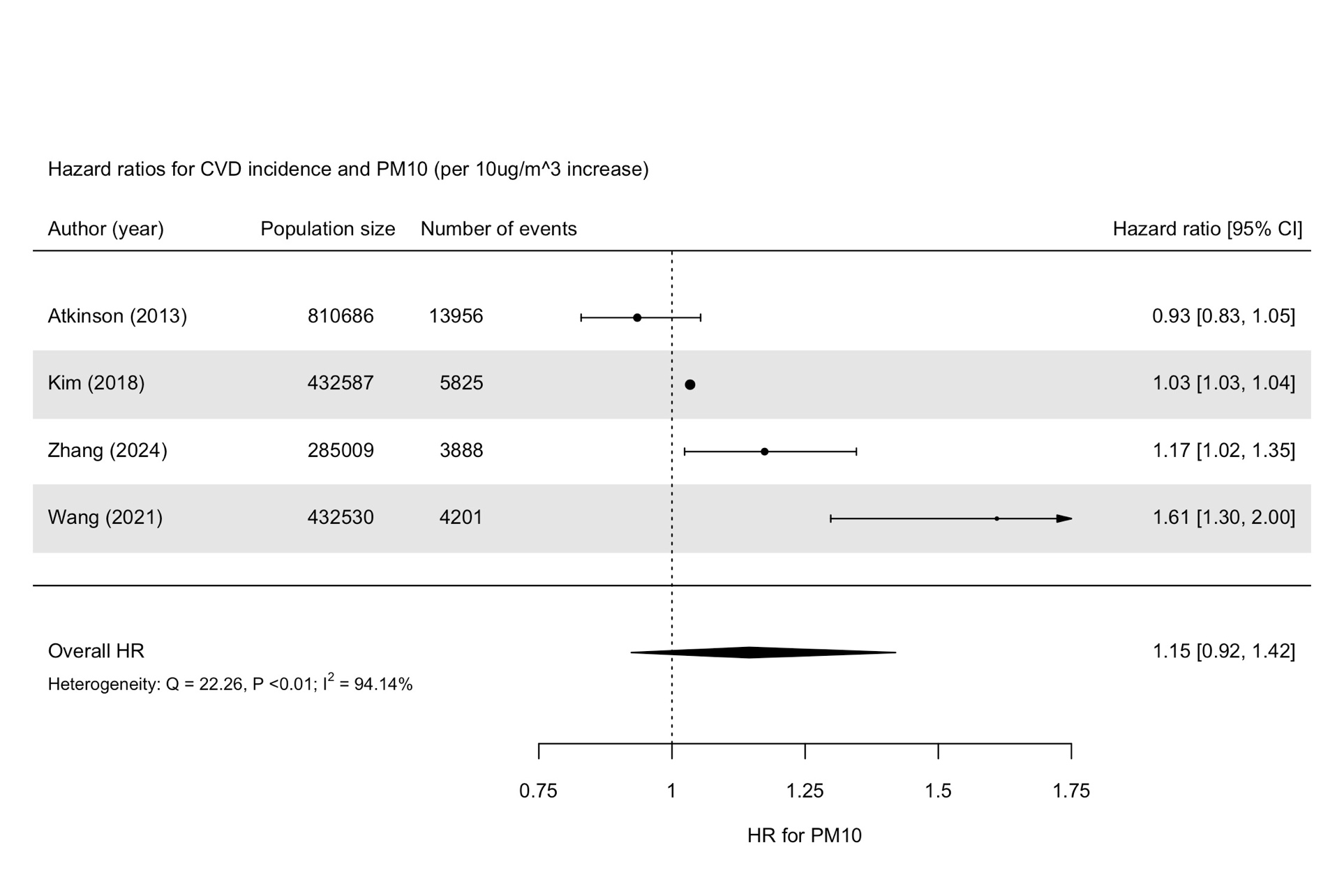
**

**Figure S5: Forest plot SO2 meta-analysis**

**
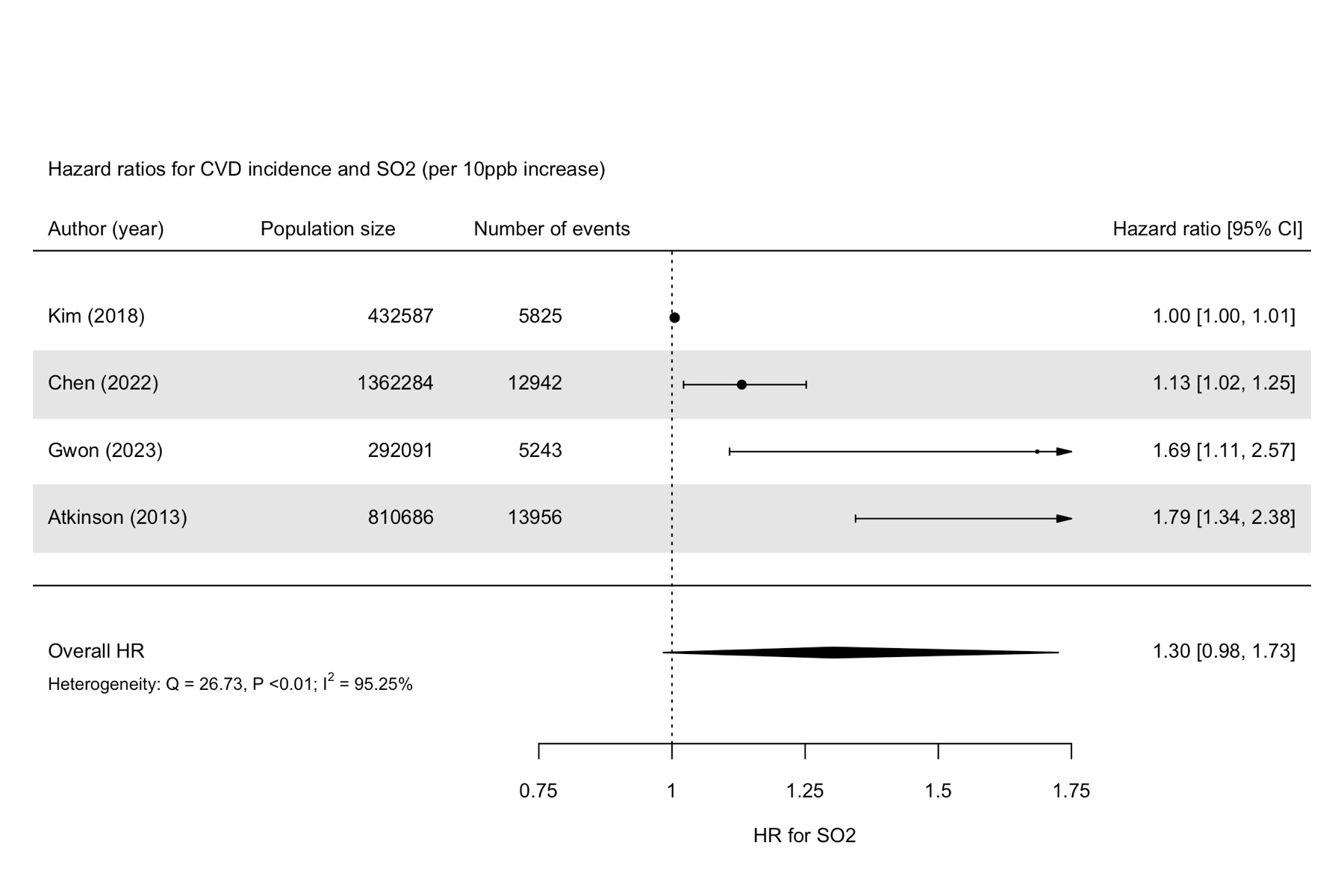
**

**Figure S6: Forest plot O3 meta-analysis**

**
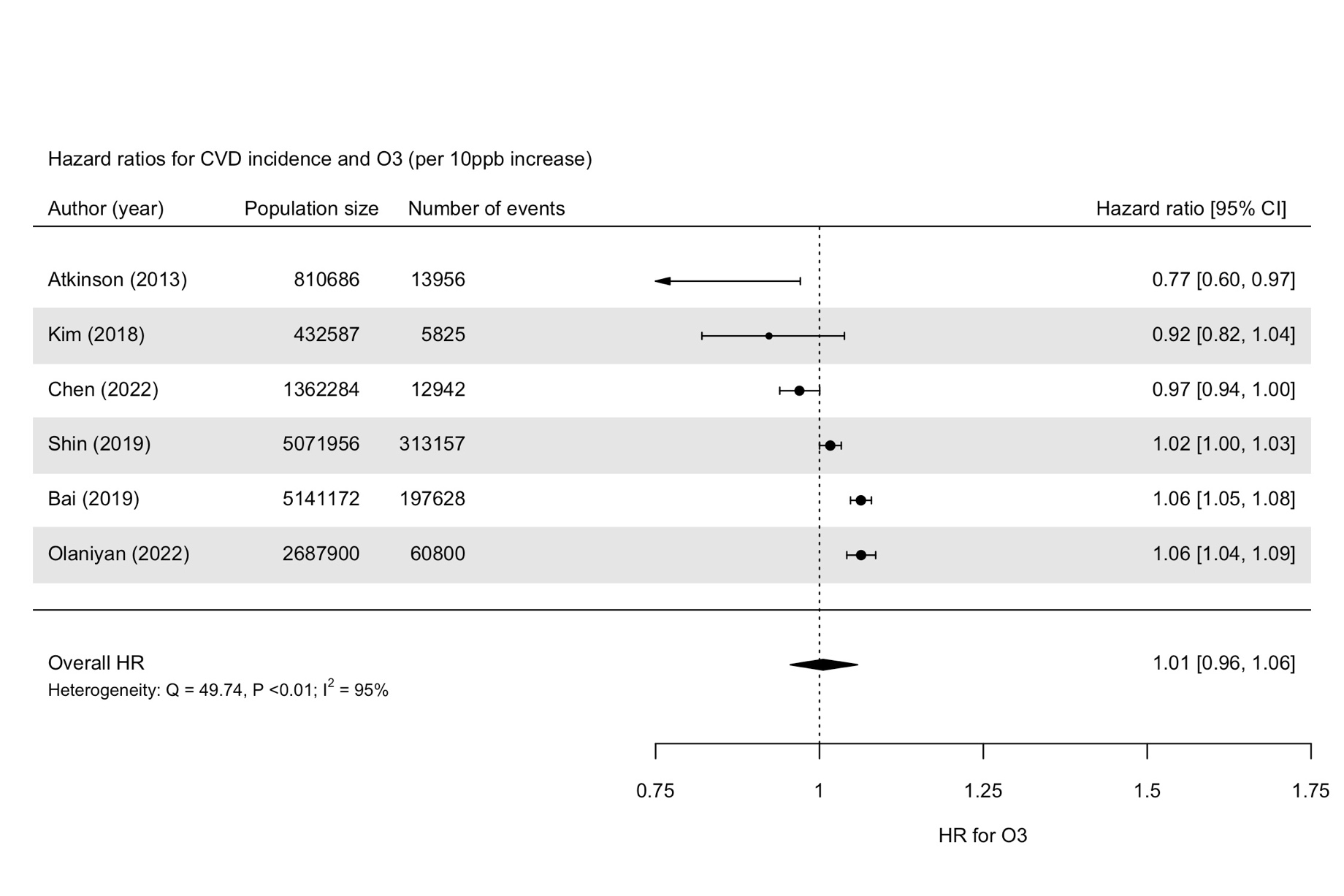
**

**Figure S7: Forest plot road-traffic noise meta-analysis**

**
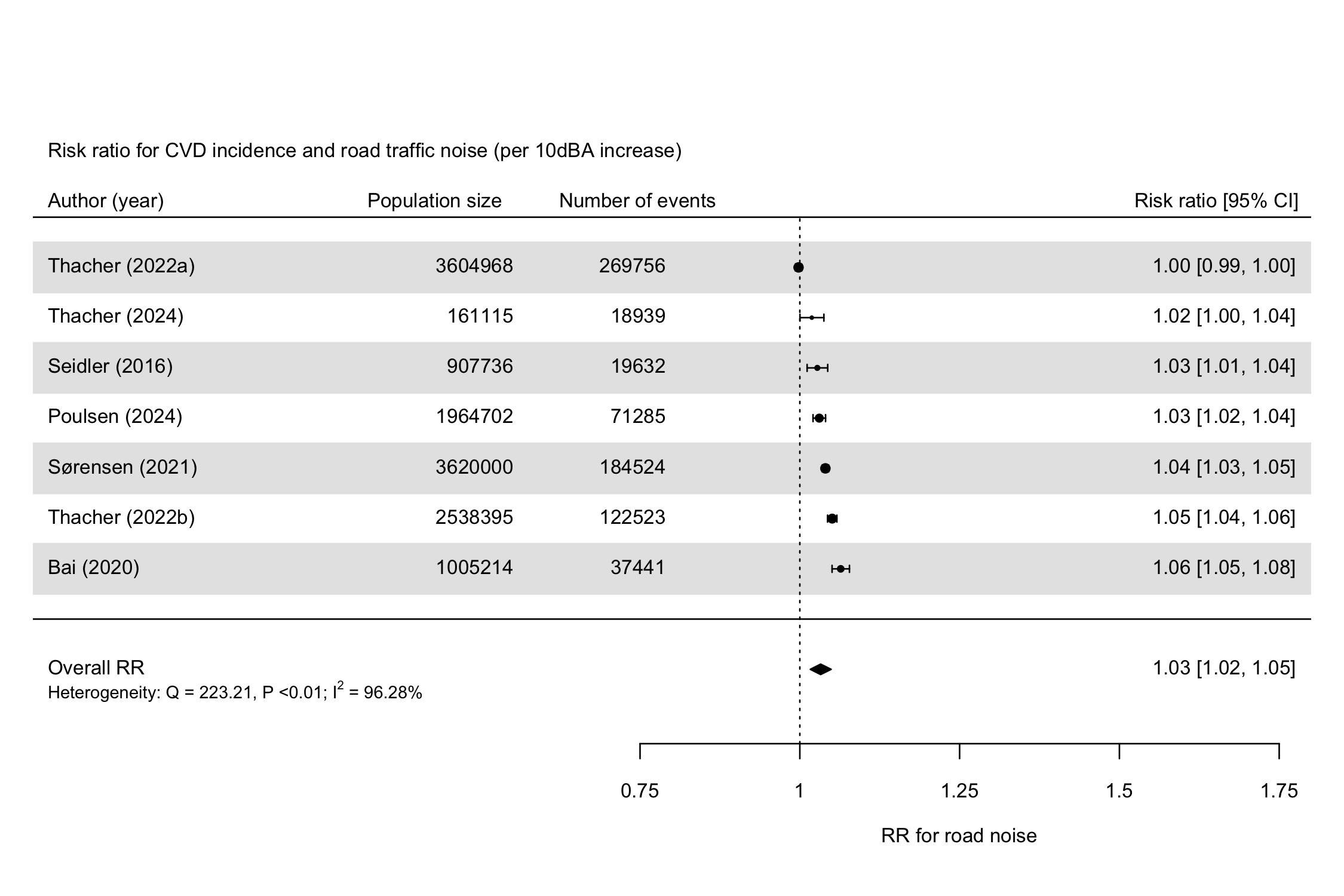
**

**Figure S8: Forest plot railway noise meta-analysis**

**
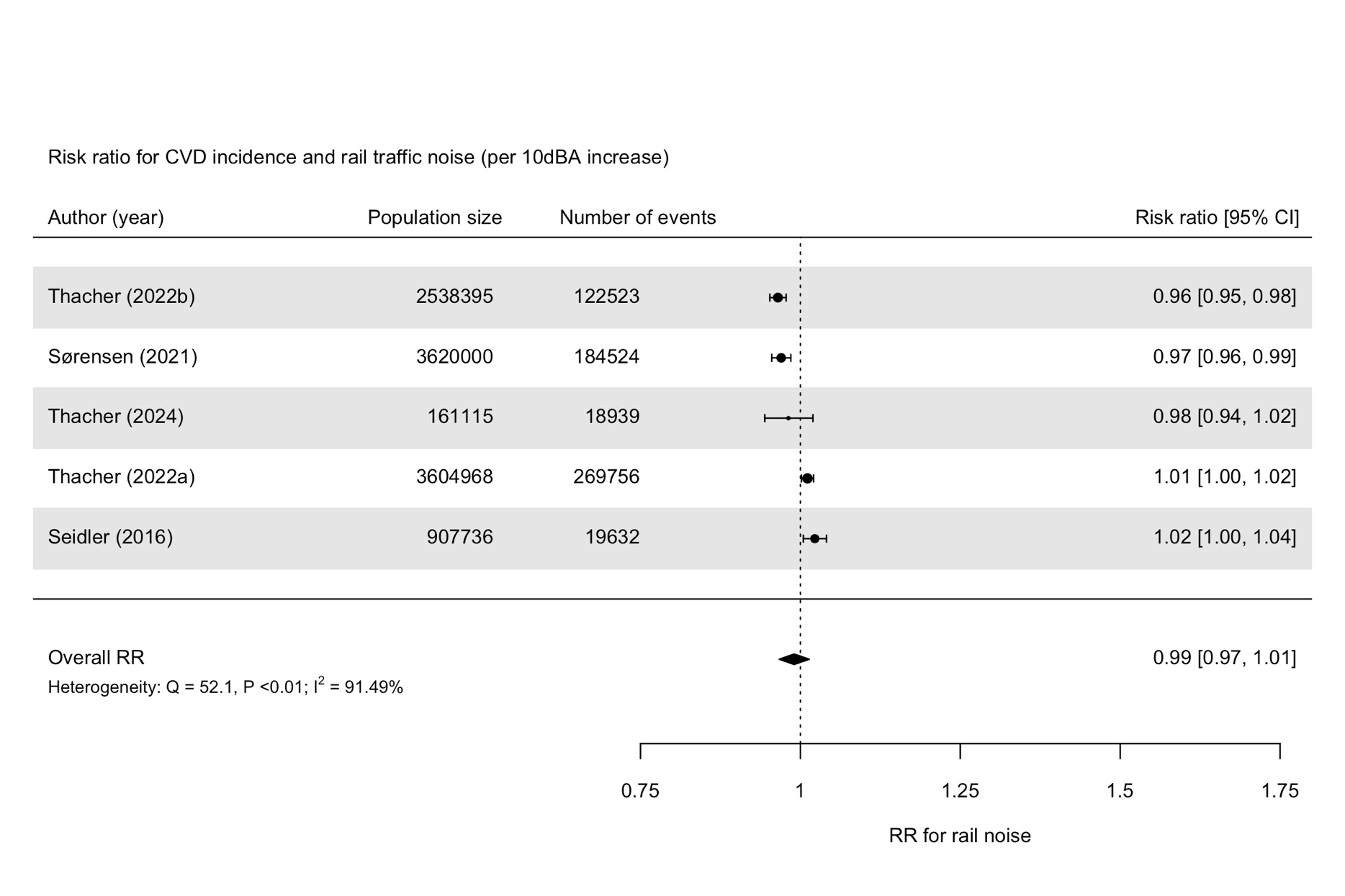
**

**Figure S9: Forest plot intermediate deprivation measure (vs low) meta-analysis**

**
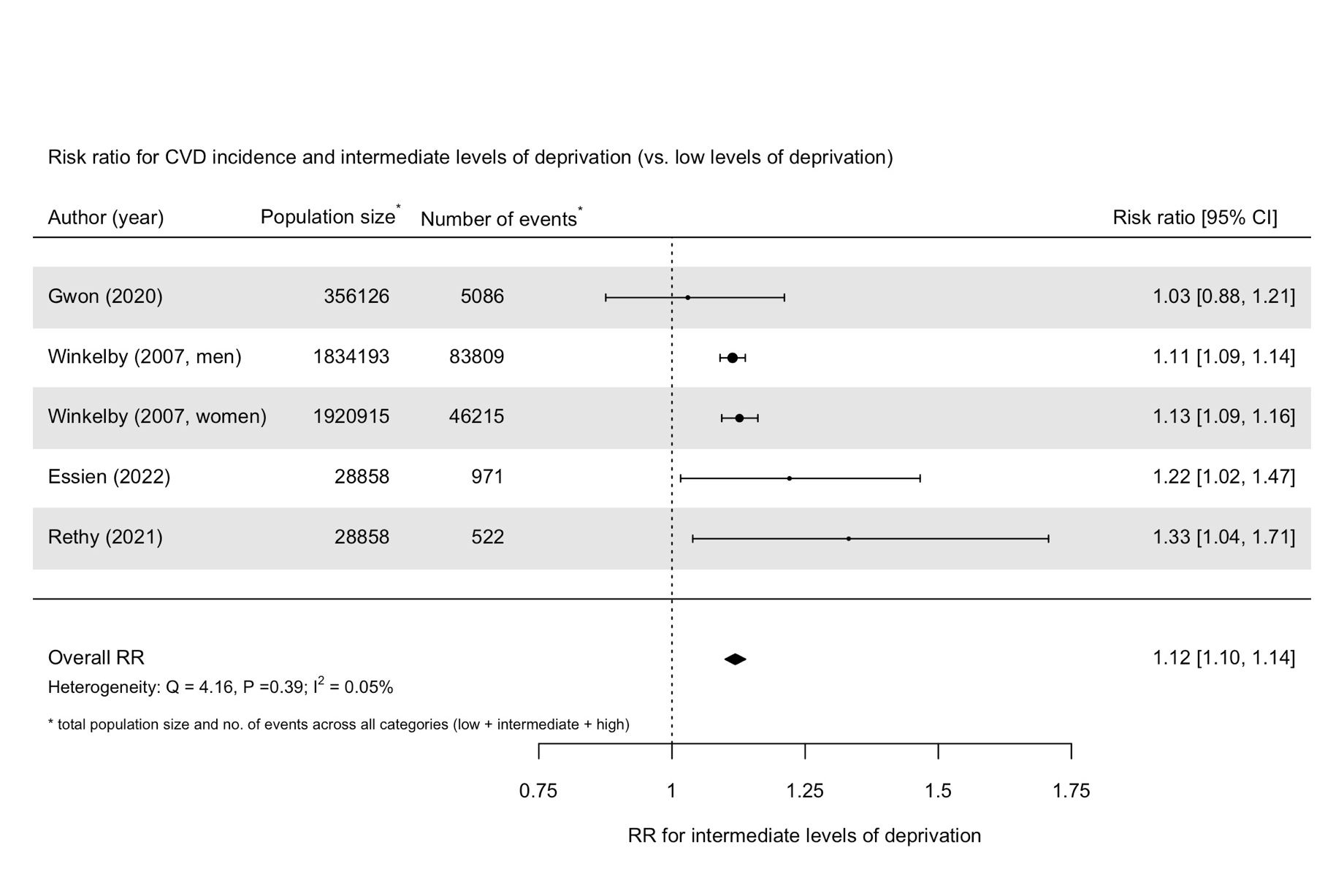
**

**Figure S10: Forest plot high deprivation measure (vs low) meta-analysis**

**
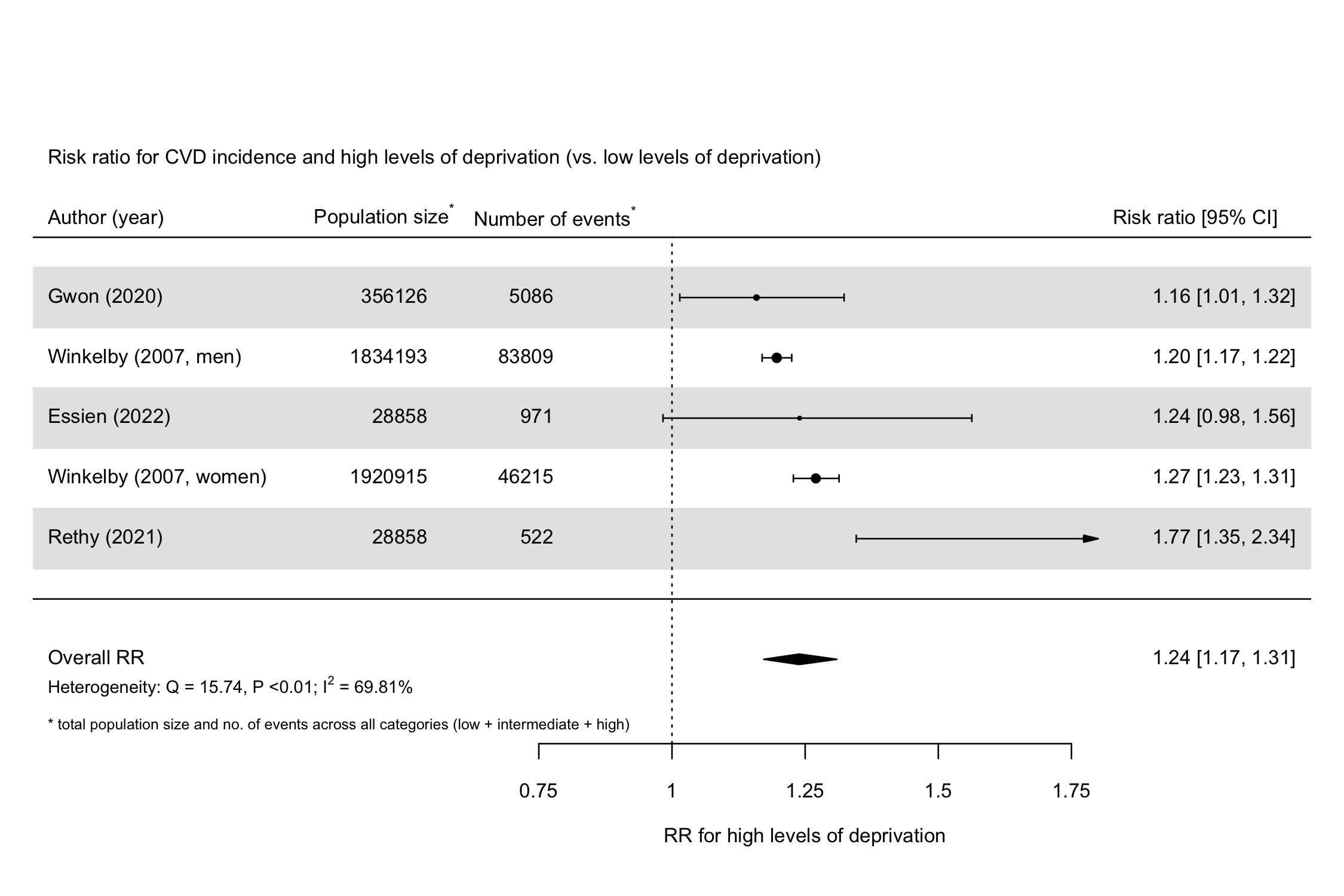
**

**Figure S11: A funnel plot for air pollutant PM2.5 including Kim et al. 2017 which was identified as an extreme outlier**

**
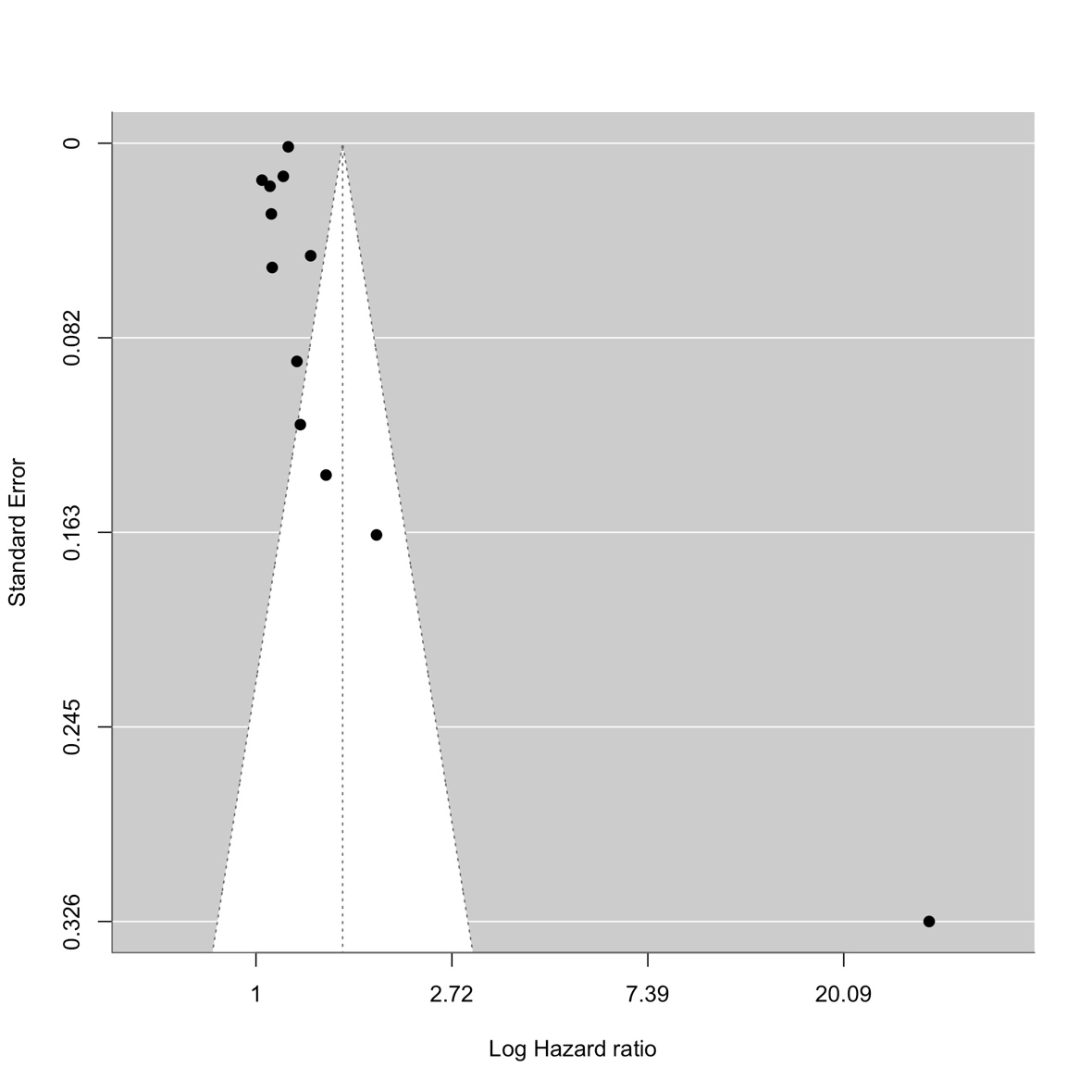
**

**Figure S12:** **Pooled meta-analysis forest plot for air pollutants including Kim 2017 (excluded as an outlier in main analysis)**

**
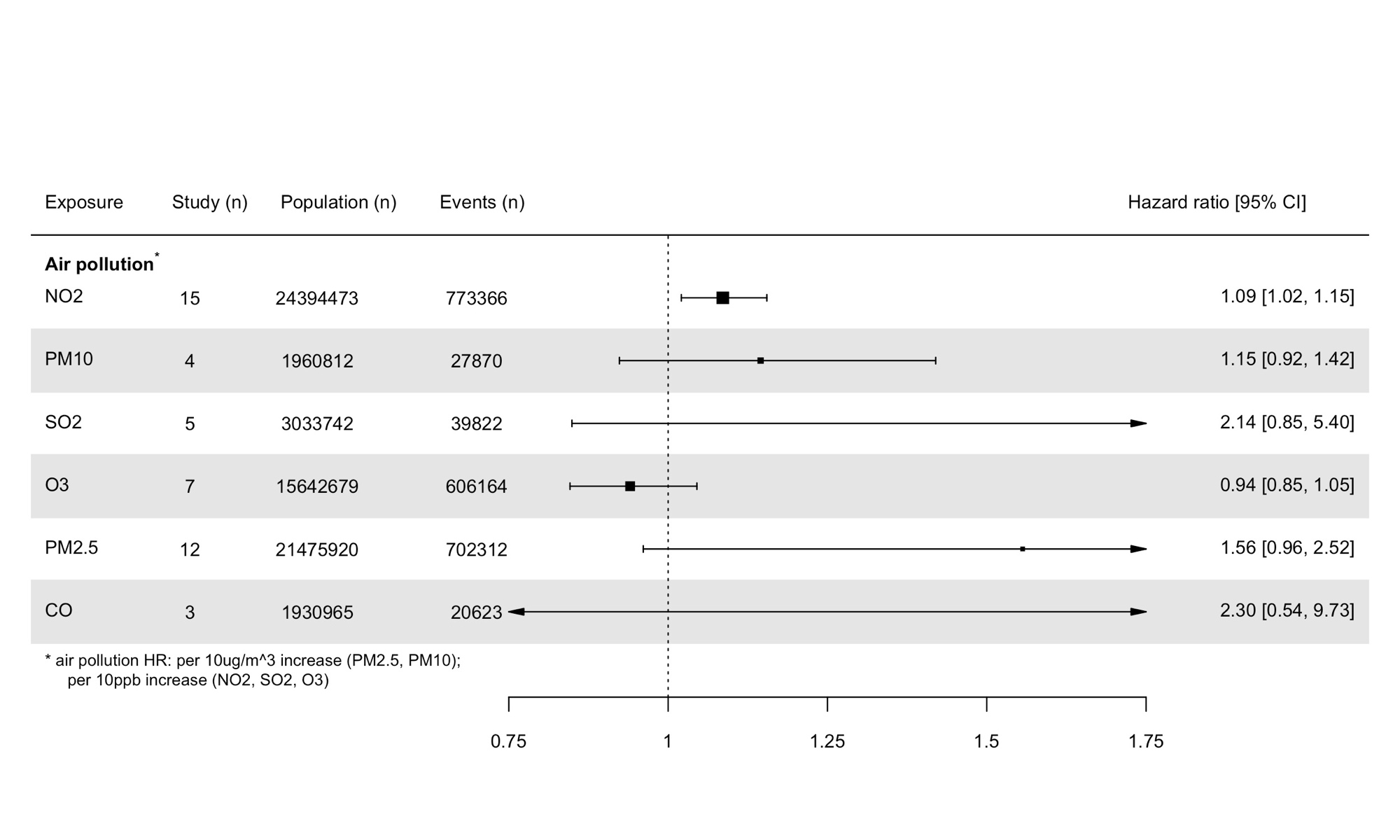
**

**Figure S13: Pooled meta-analysis forest plot excluding ‘high risk’ of bias studies**


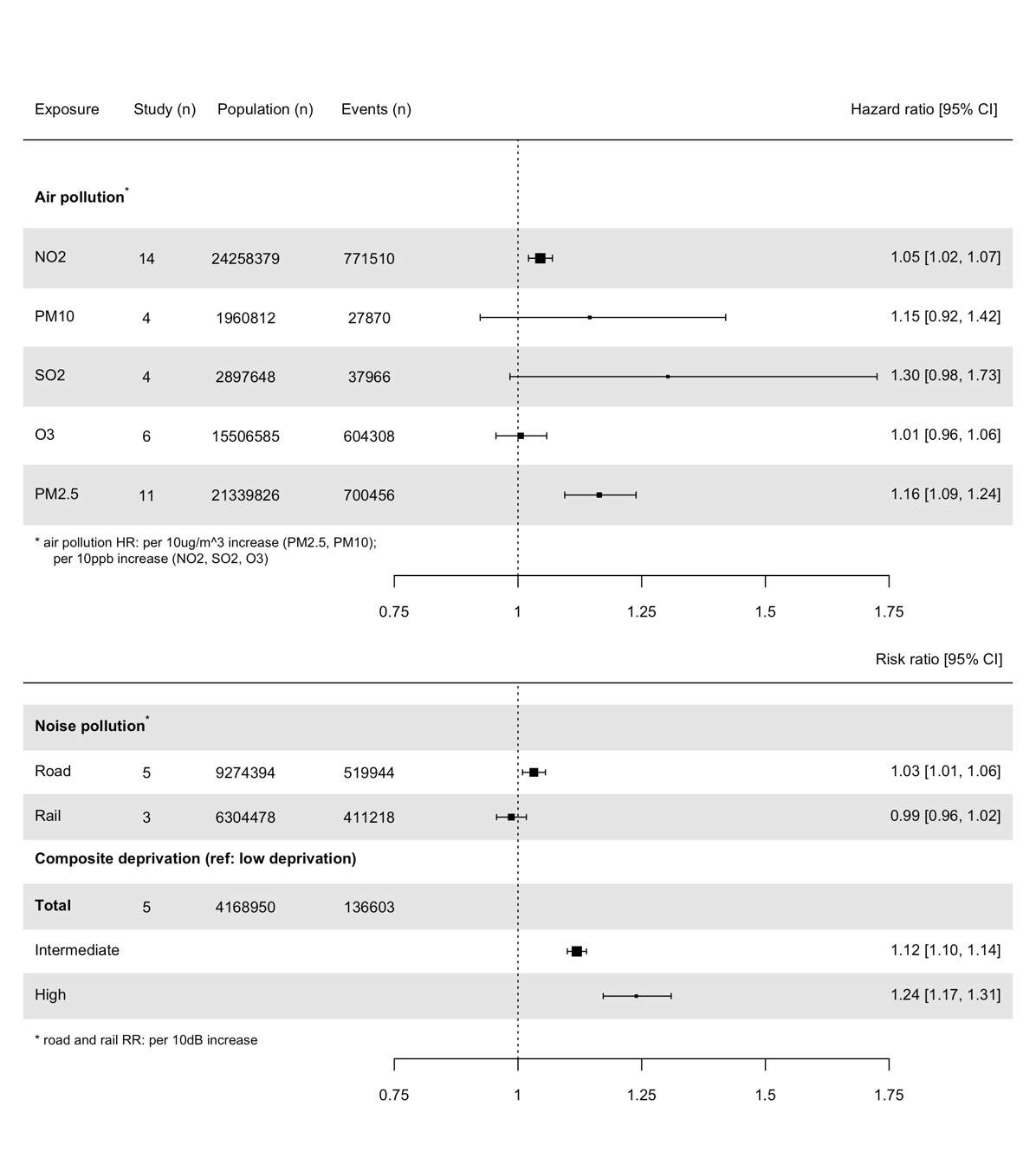


**Figure S14: Pooled meta-analysis forest plot for air pollution studies by continent**

**
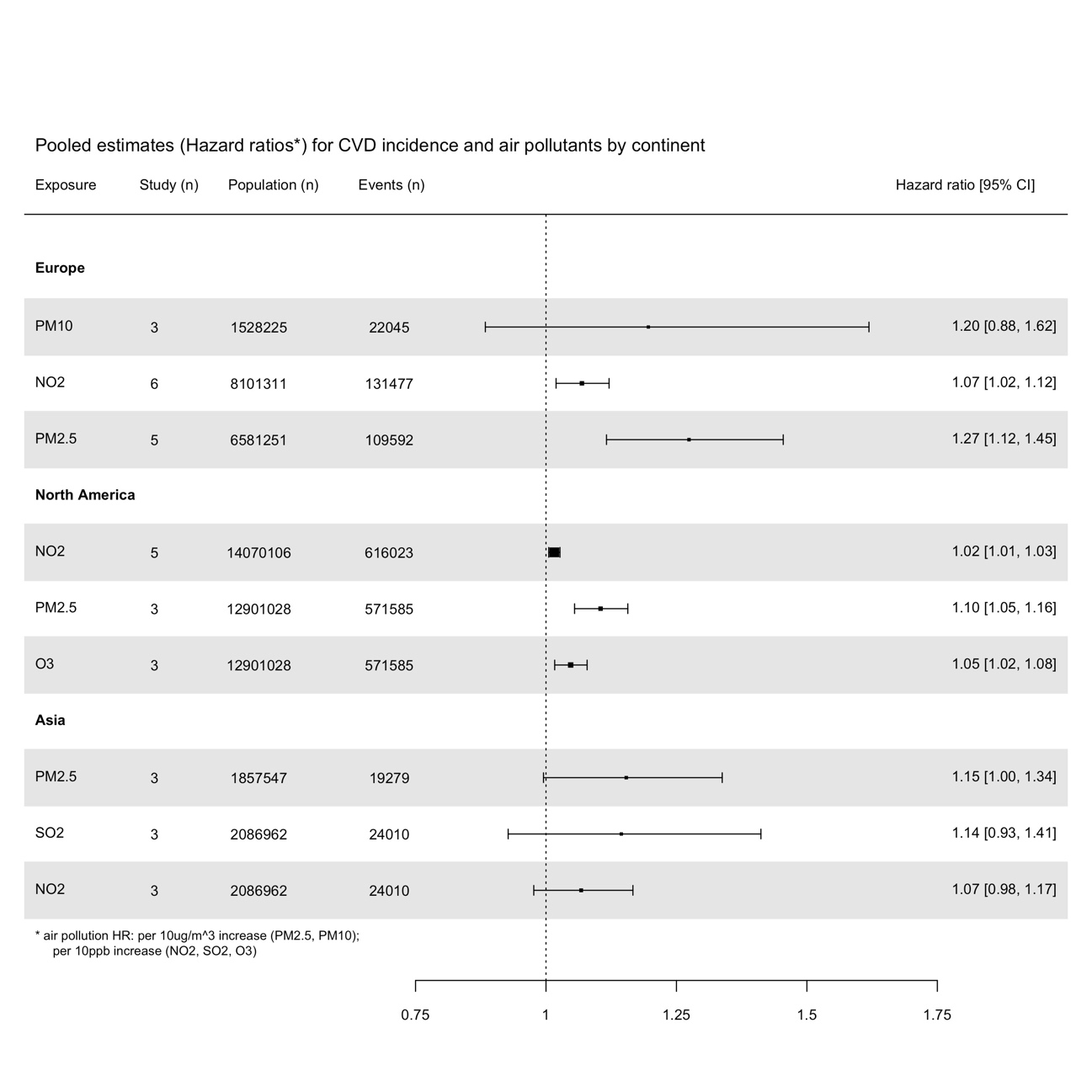
**

**Figure S15: Pooled meta-analysis forest plot for air pollution studies by publication date**

**
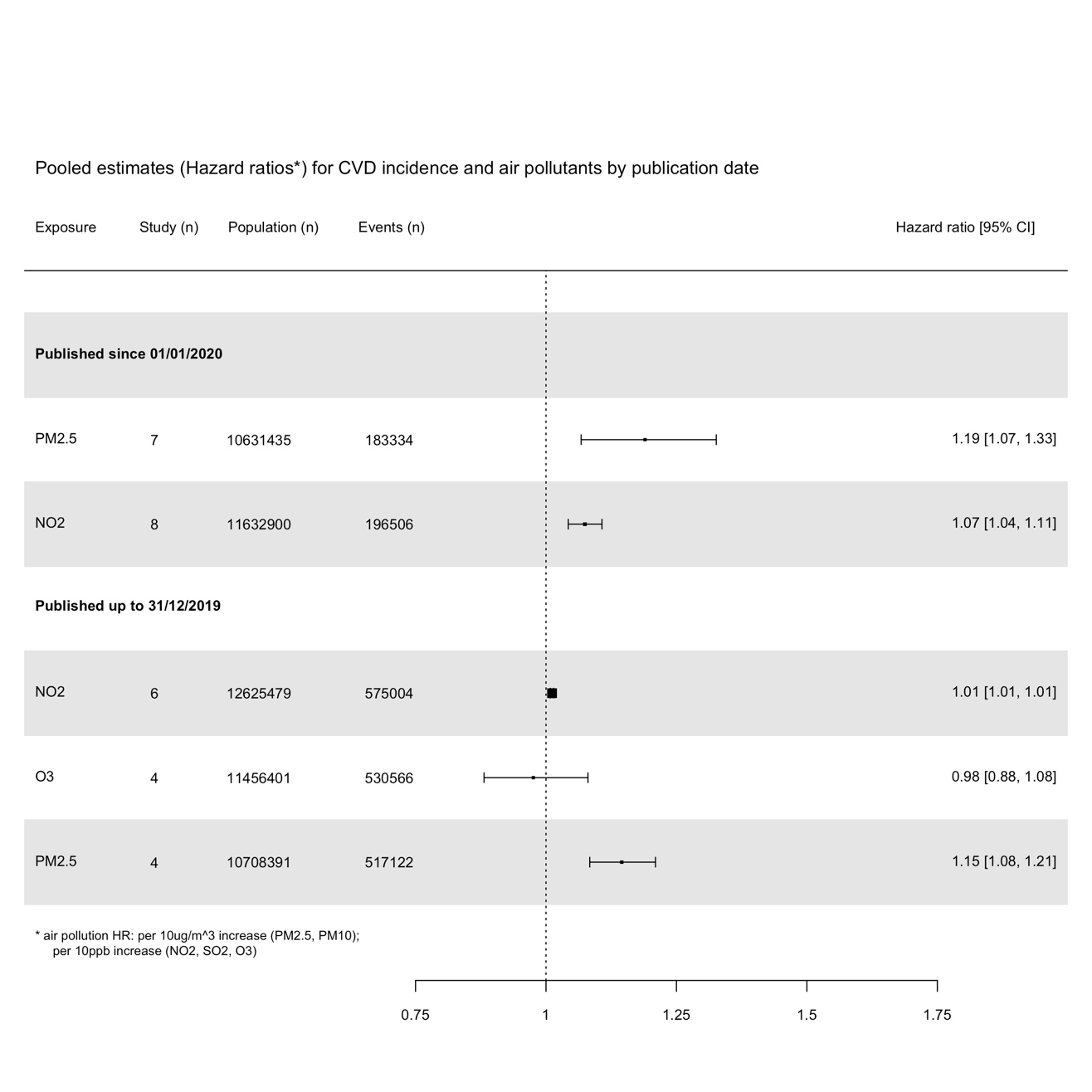
**

**Figure S16: Pooled meta-analysis forest plot for air pollution studies by sample size**

**
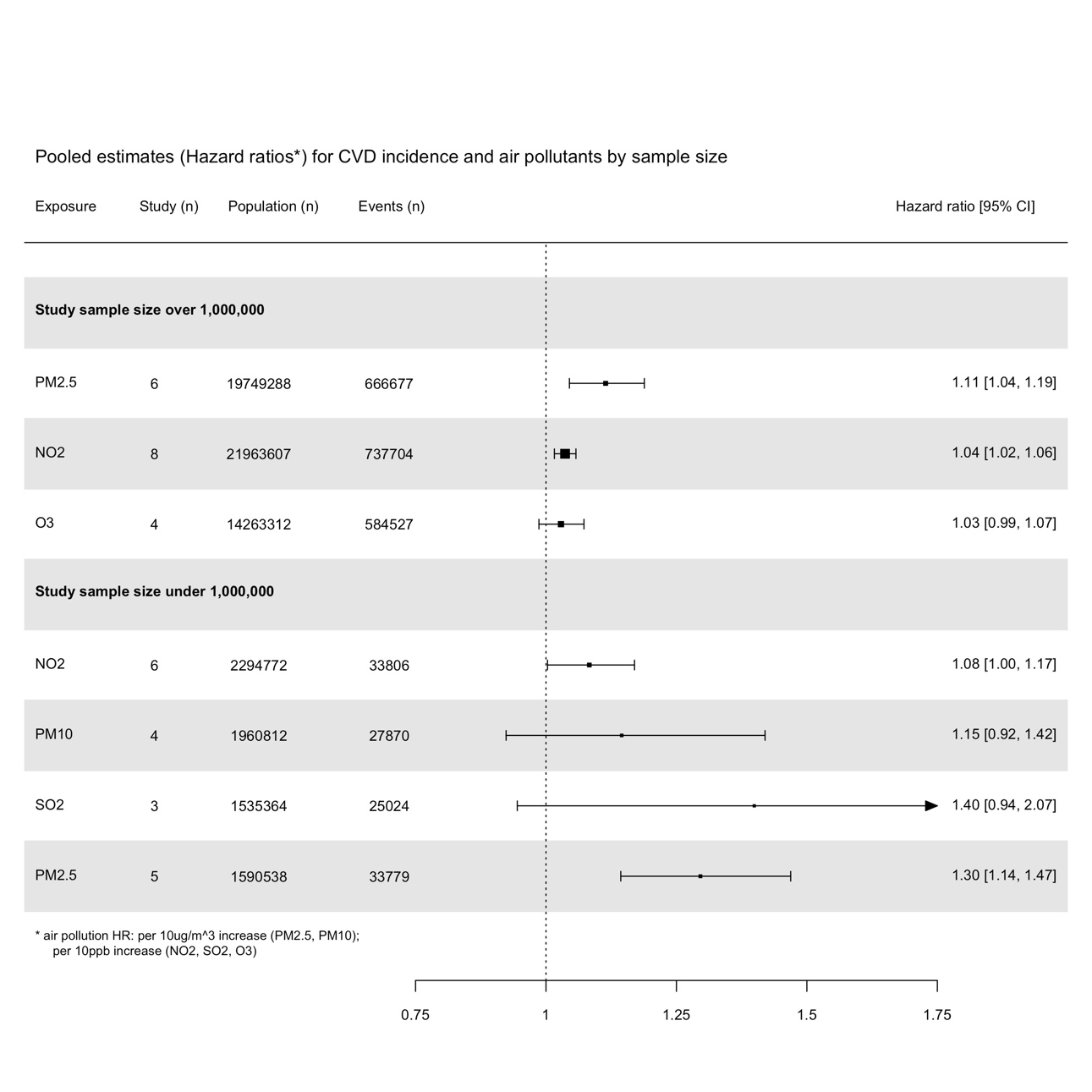
**
